# Pleiotropic and nonredundant effects of an auxin importer in *Setaria* and maize

**DOI:** 10.1093/plphys/kiac115

**Published:** 2022-03-14

**Authors:** Chuanmei Zhu, Mathew S Box, Dhineshkumar Thiruppathi, Hao Hu, Yunqing Yu, Callista Martin, Andrew N Doust, Paula McSteen, Elizabeth A Kellogg

**Affiliations:** 1 Donald Danforth Plant Science Center, St Louis, Missouri 63132, USA; 2 Department of Plant Biology, Ecology, and Evolution, Oklahoma State University, Oklahoma 74078, USA; 3 Division of Biological Sciences, Interdisciplinary Plant Group, and Missouri Maize Center, University of Missouri, Columbia, Missouri 65211, USA

## Abstract

Directional transport of auxin is critical for inflorescence and floral development in flowering plants, but the role of auxin influx carriers (AUX1 proteins) has been largely overlooked. Taking advantage of available AUX1 mutants in green millet (*Setaria viridis*) and maize (*Zea mays*), we uncover previously unreported aspects of plant development that are affected by auxin influx, including higher order branches in the inflorescence, stigma branch number, glume (floral bract) development, and plant fertility. However, disruption of auxin flux does not affect all parts of the plant, with little obvious effect on inflorescence meristem size, time to flowering, and anther morphology. In double mutant studies in maize, disruptions of *ZmAUX1* also affect vegetative development. A green fluorescent protein (GFP)-tagged construct of the Setaria AUX1 protein Sparse Panicle1 (SPP1) under its native promoter showed that SPP1 localizes to the plasma membrane of outer tissue layers in both roots and inflorescences, and accumulates specifically in inflorescence branch meristems, consistent with the mutant phenotype and expected auxin maxima. RNA-seq analysis indicated that most gene expression modules are conserved between mutant and wild-type plants, with only a few hundred genes differentially expressed in *spp1* inflorescences. Using clustered regularly interspaced short palindromic repeats (CRISPR)–Cas9 technology, we disrupted *SPP1* and the other four *AUX1* homologs in *S. viridis*. SPP1 has a larger effect on inflorescence development than the others, although all contribute to plant height, tiller formation, and leaf and root development. The AUX1 importers are thus not fully redundant in *S. viridis*. Our detailed phenotypic characterization plus a stable GFP-tagged line offer tools for future dissection of the function of auxin influx proteins.

## Teaser

Mutations in a single auxin importer gene uncover broad and unexpected effects in nearly all aspects of the development of shoots, inflorescences, and flowers.

## Introduction

The plant hormone auxin is a mobile signal that is transported between cells by both influx and efflux proteins (Naramoto, 2017). It is involved in organ initiation and growth in all parts of the plant and is particularly well known for its effects on branching (Gallavotti, 2013; Taylor-Teeples et al., 2016; Naramoto, 2017; Olatunji et al., 2017; Korver et al., 2018). Efflux proteins, particularly homologs of PIN-FORMED1 (PIN1; Petrásek et al., 2006; Balzan et al., 2014; Naramoto, 2017), have been studied extensively in many plant species, with particular attention in Arabidopsis (*Arabidopsis thaliana*), long the model of choice for studies of auxin function. As a result, much has been discovered about the flow of auxin out of cells (e.g. Verna et al., 2019) and how auxin gradients are established throughout the plant (e.g. Heisler et al., 2005; Wang and Jiao, 2018 and many others).

In contrast, the flow of auxin into cells (auxin influx) has received much less attention, particularly in reproductive organs. In Arabidopsis single-gene mutants of any of the four auxin influx carriers (AUXIN1 [AUX1] and LIKE AUXIN1 [LAX1–3]) have normal above-ground structures and higher order mutants affect only leaf phyllotaxis (Kleine-Vehn et al., 2006; Bainbridge et al., 2008; Peret et al., 2012; Swarup and Péret, 2012). Perhaps, because of this subtle mutant phenotype, far less is known about influx than efflux, especially as regards vegetative and inflorescence development. Also the *AUX1/LAX* genes in Arabidopsis are more closely related to each other than any of them is to the *AUX1-like* genes known in grasses (Huang et al., 2017). This lack of one-to-one correspondence, in addition to the lack of a strong phenotype in Arabidopsis, prevents direct extrapolation from Arabidopsis to any monocot, particularly cereal crops and their relatives.

A recently identified mutation in an auxin influx carrier in the model grass green millet (*Setaria viridis*), SPARSE PANICLE1 (SPP1) (Huang et al., 2017), offers an opportunity to uncover aspects of auxin influx disruption. SPP1 is homologous to the maize (*Zea may*s) protein ZmAUX1 and to the four Arabidopsis AUX1 proteins, but unlike in Arabidopsis, the *spp1* mutation (presumed to abolish gene function) causes an obvious defect in the inflorescence, thus providing a system in which the effects of disrupting influx are easily seen. *SPP1* was named for the wide spacing of its primary inflorescence branches, and its role in auxin transport was supported by observation of clearly agravitropic roots (Huang et al., 2017). However, few other aspects of plant growth and development were considered in the original paper, including many that would be expected to require normal auxin transport. For example, the *S. viridis* inflorescence typically exhibits many orders of branches, some of which produce spikelets and others that end blindly (known as bristles; see Doust and Kellogg (2002)). Disruption of SPP1 should affect these higher order branches and the balance of spikelet-bearing branches and bristles, as well as other aspects of above-ground architecture such as tillering and relevant gene expression.

AUX1 mutants have been reported in other grasses (maize, rice [*Oryza sativa*], and purple false brome [*Brachypodium distachyon*]) but these studies focused on roots (Yu et al., 2015; Zhao et al., 2015; Huang et al., 2017; van der Schuren et al., 2018), which were agravitropic in all species, consistent with disruption of auxin pathways. In addition, the rice mutants had fewer lateral roots (Yu et al., 2015; Zhao et al., 2015), whereas the *S. viridis* mutants had a normal number (Yu et al., 2015; Zhao et al., 2015; Huang et al., 2017; van der Schuren et al., 2018). Neither Yu et al. (2015) nor Zhao et al. (2015) reported changes in the inflorescence in rice *OsAUX1* mutants. In *Brachypodium distachyon*, *bdaux1* mutants are sterile and some above-ground structures are affected, but the phenotypes are not described in detail (van der Schuren et al., 2018). Thus the role of AUX1 in above-ground development remains largely unexplored, especially in grasses and cereal crops.

Here we show that mutations in *SPP1* (=*SvAUX1*) and its homolog in maize affect shoot phenotypes including development of the gynoecium and floral bracts (glumes); these are not side-effects of meristem size variation or differences in developmental timing. Based on the phenotypes of higher order mutants involving all five *S. viridis AUX1-like* loci, we show that *SPP1* is not redundant with the other loci and is the major locus controlling inflorescence architecture. *ZmAUX1*, investigated because of the wealth of auxin-related mutants in maize, enhanced the mutant phenotypes of several auxin pathway genes and revealed an unexpected enhanced effect on leaf number. In *S. viridis*, SPP1 was internally tagged, and localized to the plasma membrane (PM) of epidermal cells in inflorescence branch meristems (BMs) and roots. Only a few hundred genes, including several known to be involved in inflorescence development, are differentially expressed between *spp1* and wild-type (WT) inflorescences, indicating highly specific changes in the transcriptome.

## Results

### *spp1* affects tillering, inflorescence branching, gynoecium development, and root hair formation

**Figure 1:**
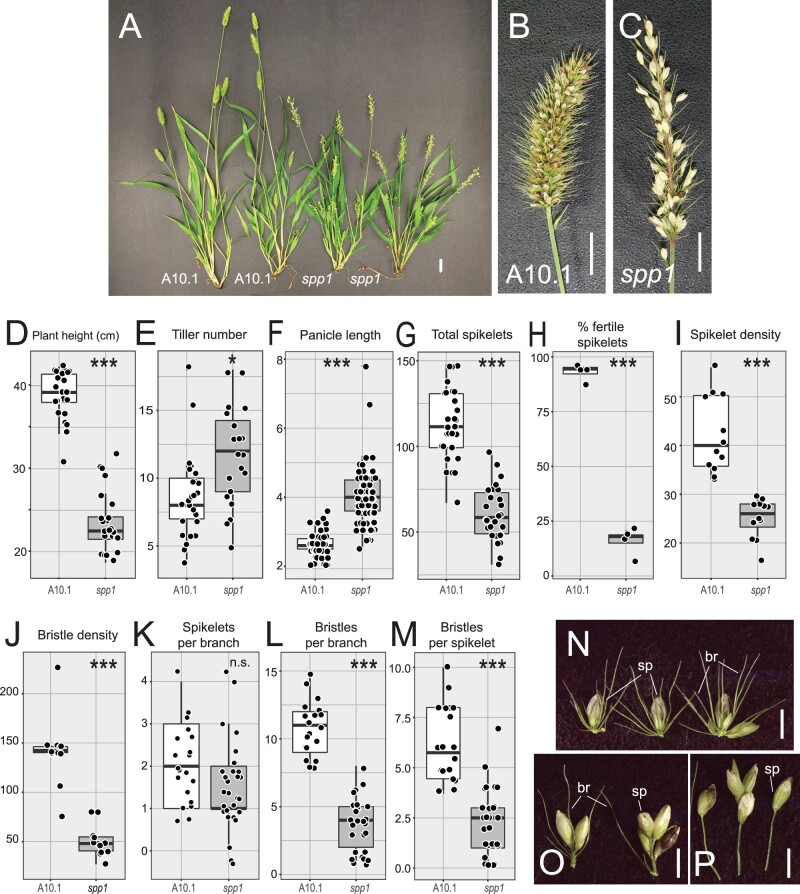
Phenotypes of *spp1* mutants. A, Mature plants of WT (A10.1, left) and *spp1* mutants (right) at 22 DAS. Scale = 2 cm. B and C, Mature panicles. B, WT, (C) *spp1*. Scale = 1 cm. Brown or black spikelets contain fully developed seeds, whereas whitish spikelets are often infertile. D–M, Comparisons of trait values between WT (left box, white) and *spp1* mutant (right box, gray) plants. Boxes extend from lower quartile boundary to upper quartile; horizontal bar is median. Whiskers extend to the smallest and largest values within 1.5 times the interquartile range. Dots indicate individual data points. Significance values determined by Welch’s *t* test. Square, 0.01–0.05, *P <0.01, **P <0.001, ***P <0.0001. D, Plant height (cm), (E) Tiller number, (F) Panicle length (cm), (G) Total number of spikelets, (H) Percent fertile spikelets, (I) Spikelet density (number of spikelets per cm), (J) Bristle density (number of bristles per cm), (K) Spikelets per primary branch, (L) Bristles per primary branch, (M) Bristles per spikelet (values from K divided by values from L), (N–P) Individual primary branches from WT (N) and *spp1* (O and P) mutants. Scale = 2 mm. sp, spikelet, br, bristle. Mean, standard deviation (sd), sample sizes, and *P*-values in Supplemental Table S1.

Mutations in SPP1 affect many aspects of plant development having to do with growth and branching (Figure 1; Supplemental Table S1). In addition to the eponymous sparse panicle phenotype (Figure 1, A–C), mutant plants were significantly shorter than WT (Figure 1, A and D) and produced more tillers (Figure 1, A and E). Mutant panicles were significantly longer than WT (Figure 1, B, C, and F), but increased length did not result in higher yield. Instead, mutants had fewer spikelets at maturity (Figure 1G) and fewer of these were fully developed and fertile (Figure 1H). The reduced number and fertility of spikelets was not caused by a developmental delay; the transition to reproductive growth and flowering in *spp1* mutant plants was only slightly later than in A10.1 (Supplemental Figure S1, A and B), and barely statistically significant. Fertile florets (upper lemma + palea) were significantly larger in the mutant (Supplemental Figure S1C) but percent germination did not differ (Supplemental Figure S1D). Culms (peduncles) were generally thinner in the mutant but overall culm anatomy was similar (Supplemental Figure S1, E–G).

The lower density of spikelets and bristles (fewer of each per cm; Figure 1, I and J) could reflect reduced density of primary branches (observed in early development; see comments on SEM data below) and/or a change in the numbers of spikelets and bristles per branch; the latter would indicate an effect of the mutation on secondary and higher order branches. In mutant panicles, the primary branches have about the same number of spikelets as in WT (Figure 1, K, N, and O), but significantly fewer bristles (Figure 1, L and N–P) and therefore a lower ratio of bristles to spikelets (Figure 1, M–P). In addition, ∼15% of branches in *spp1* had one or a few spikelets at the terminus of a long branch without additional bristles, compared to <1% of A10.1 branches (Figure 1P). Together these observations suggest that the *spp1* mutation affects both the formation of higher order branches and the specification of those branches as spikelets or bristles.

**Figure 2:**
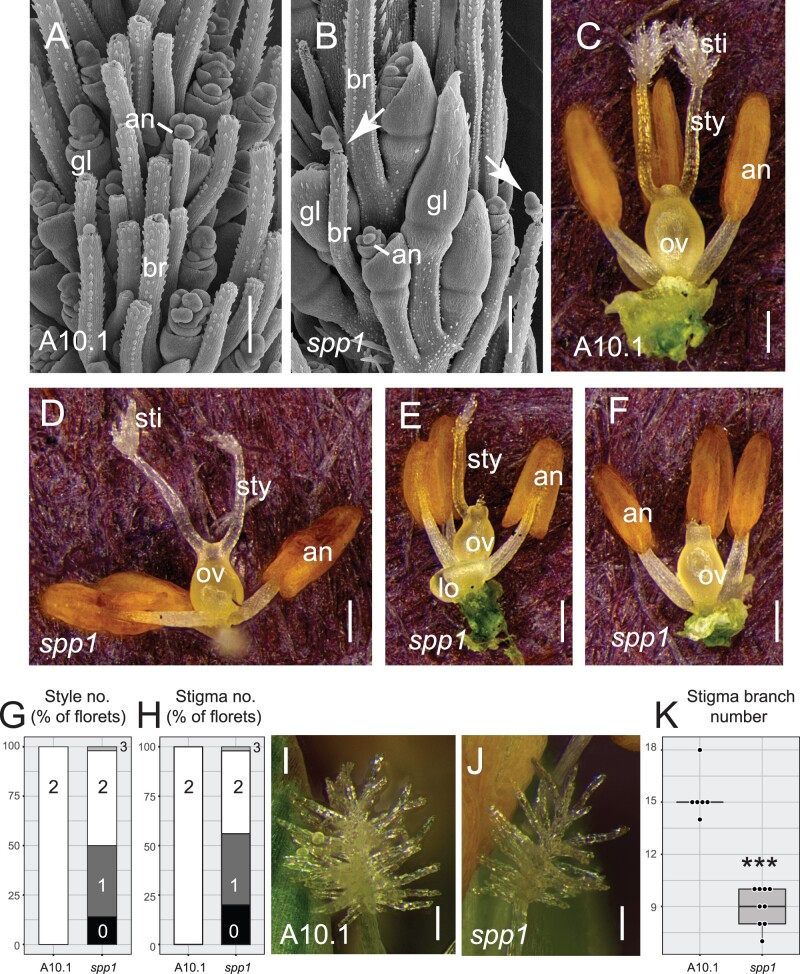
Floral phenotypes of *spp1*. A and B, SEM images of developing spikelets and bristles at 18 DAS. A, WT (A10.1), (B) *spp1*. Arrows show un-detached meristems on bristle tips. Scale = 200 µm. C–F, Reproductive organs in WT (C) and *spp1* (D–F) mutant florets, showing abnormal development of stigmas and styles in the mutants. Scale = 250 μm. G and H, Bar graphs showing percentage of florets with 0, 1, 2, or 3 styles (G) and stigmas (H) in WT (left) and *spp1* (right). I and J, Stigmas from WT (I) and *spp1* florets (J). Scale = 100 μm. K, Stigma branch number counted from one side of the stigma on the focal plane in WT (left box, white) and *spp1* (right box, gray) plants. Box plots as in Figure 1. Significance values determined by Welch’s *t* test and symbols as in Figure 1. Mean, sd, sample sizes, and *P*-values in Supplemental Table S1. an, anther; br, bristle; gl, glume; lo, lodicules; ov, ovary; sti, stigma; sty, style. Image (A) reproduced with permission from Zhu et al. (2018).

Floral morphology and early development are affected in *spp1* mutants and are likely to be at least partially responsible for the fertility defects of the mutant (Figure 2; Supplemental Table S1). At 18 d after sowing (DAS) when the anthers and gynoecium were first visible in both A10.1 and *spp1*, glumes in the WT were shorter than the flowers (Figure 2A), whereas those in the mutants were unusually long, nearly enclosing the flowers (Figure 2B). In addition, the mutants had fewer branches, bristles, and spikelets at this stage, consistent with the reduced number of bristles per spikelet at maturity (Figure 1M). All spikelets in both genotypes had the expected number of glumes (two) and florets (two), with lemmas, paleas, lodicules, and stamens developing apparently normally in both mutant and WT plants (Figure 2, C–F).

Gynoecium formation was abnormal in *spp1*. Only about half of the florets of each mutant plant have two styles, the normal number in WT (Figure 2, C–G). Although recorded as categorical for simplicity, reduction in style and stigma number was in fact quantitative and asymmetrical. One style could be substantially shorter than the other, or reduced to a small protrusion, or missing altogether; in two cases (on different plants), an extra protrusion led to three style-like structures total. If two styles were present, often only one would have a well-developed stigma. Only in one floret of the 50 examined were both styles and stigmas reduced equally and symmetrically. Stigmas in *spp1* plants, when present, were significantly less branched than in WT (Figure 2, H–K; Supplemental Table S1).

Neither primary root length nor lateral root number was obviously altered in *spp1* (Supplemental Figure S1N), but root hair density was significantly lower on both primary and lateral roots in *spp1* compared to A10.1 (Supplemental Figure S1, H, I, L, and M; Supplemental Table S1). In addition, the distance from root tip to the first root hair initiation site was significantly longer in mutant roots (Supplemental Figure S1, J and K).

By applying synthetic auxins to roots, we showed that SPP1 could potentially function in auxin import. In response to a mock auxin treatment, *spp1* roots were agravitropic (Supplemental Figure S2, A and B), as reported (Huang et al., 2017), and had fewer root hairs than WT (Supplemental Figure S2, G and H). 2,4-Dichlorophenoxyacetic acid (2,4-D), which requires auxin importer proteins to move into the cells, could not rescue the mutant phenotypes in roots, consistent with our hypothesis that SPP1 is a bona fide auxin importer (Supplemental Figure S2, C, D, I, J, and M). In contrast, the lipophilic auxin 1-Naphthaleneacetic acid (NAA), which can diffuse freely across the PM, restored both the gravitropic response of *spp1* roots (Supplemental Figure S2, E and F) and also the normal density of root hairs (Supplemental Figure S2, K–M).

### SPP1 controls inflorescence branch initiation, elongation, and identity, but not meristem size

**Figure 3:**
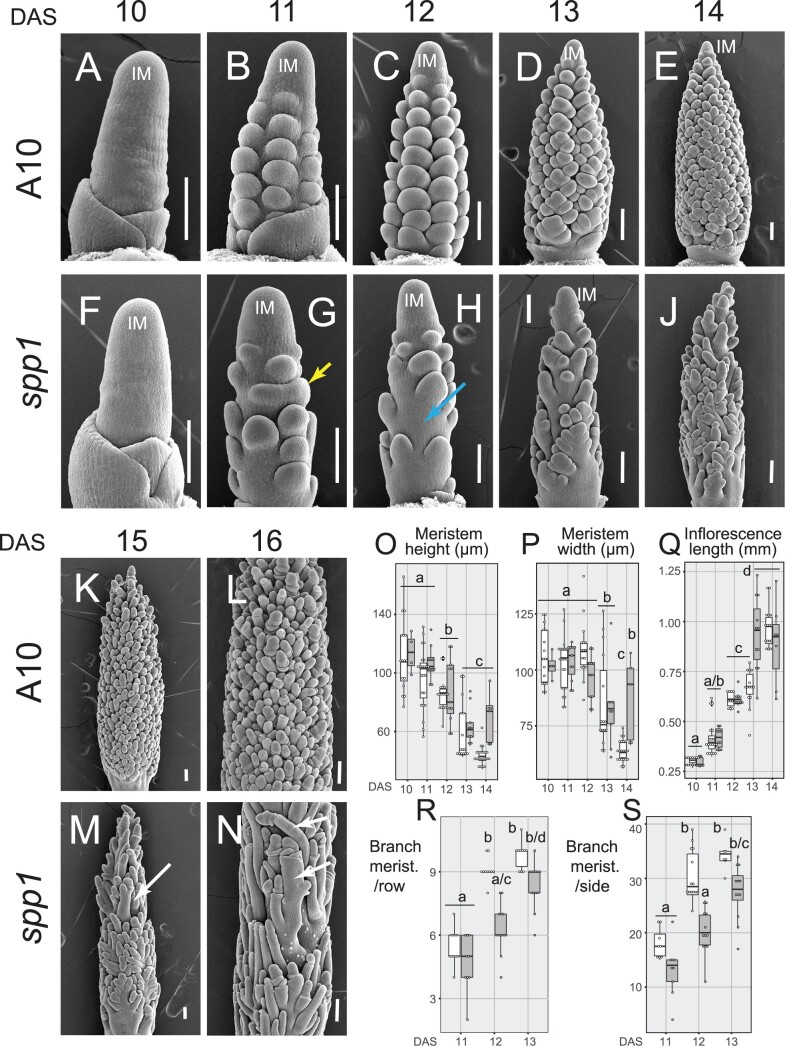
Early inflorescence development of *spp1*. A–N, SEM images of WT (A10.1) (A–E, K, and L) and *spp1* (F–J, M, and N) inflorescences at 10–16 DAS (left to right, one picture for each stage, respectively). Yellow arrow, fused primary BMs; blue arrow, failed initiation of primary BM; white arrows, elongated branch primordia. (O–S) Comparisons of WT (white) and *spp1* (gray) inflorescences as measured from SEM photos. O, meristem height and (P) meristem width (µm) at 10–14 DAS. Q, Inflorescence length (mm) at 10–14 DAS. R and S, Number of primary BMs per vertical row (R) and the total number visible from one side of the inflorescence (S). Box plots as in Figure 1. Significance values determined by ANOVA and Tukey’s honestly significant difference (HSD) test. Boxes with the same letter are not significantly different at *P* < 0.05. Mean, sd, sample sizes, and *P*-values in Supplemental Table S2.

To explore whether the sparse panicle phenotype in *spp1* resulted from branch initiation defects linked to abnormal meristem size, we imaged early inflorescence development with scanning electron microscopy (SEM) (Figure 3, A–N; Supplemental Table S2). Meristem height (the vertical distance from the uppermost branch primordium to the apex of the meristem) dropped significantly between 11 and 12 DAS and again between 12 and 13 DAS, but WT and mutant inflorescences did not differ at any stage of development (Figure 3O). Meristem width was unchanged in either genotype over 10–12 DAS, then dropped significantly in both genotypes between 12 and 13 DAS (Figure 3P); by 14 DAS, *spp1* inflorescences were wider than those in WT (Figure 3P). Overall length of inflorescences before 14 DAS scarcely differed between *spp1* and WT (Figure 3, A–N and Q), indicating that the length difference at maturity was established later in development and probably reflected rachis elongation rather than branch initiation. By 12 DAS, primary branch number in *spp1* was significantly lower than in A10.1, whether counting branches per vertical row (Figure 3R), or all visible branches on one side of the inflorescence (Figure 3S). In contrast to A10.1, which produced primary BMs in a spiral pattern around the inflorescence meristem (IM; Figure 3, A–E, K, and L), *spp1* often failed to initiate a BM or produced unusually large primary BMs (Figure 3, F–J, M, and N). While primary BMs produced distichous secondary BMs in A10.1 (Figure 3, C–E, K, and L), secondary branches often initiated asymmetrically in *spp1* (Figure 3, H–J, M, and N).

*spp1* was defective in branch elongation and meristem fate determination. Branch primordia in *spp1* elongated more than those in A10.1 (Figure 3, A–N). While most bristles in A10.1 had lost their meristematic tip completely by 16 DAS (Figure 3L), bristles often retained their meristem in *spp1* (Figure 3N) even at 18 DAS (Figure 2, A and B).

### The *Spp1* ortholog in maize, *ZmAux1*, enhances effects of auxin-related genes

**Figure 4:**
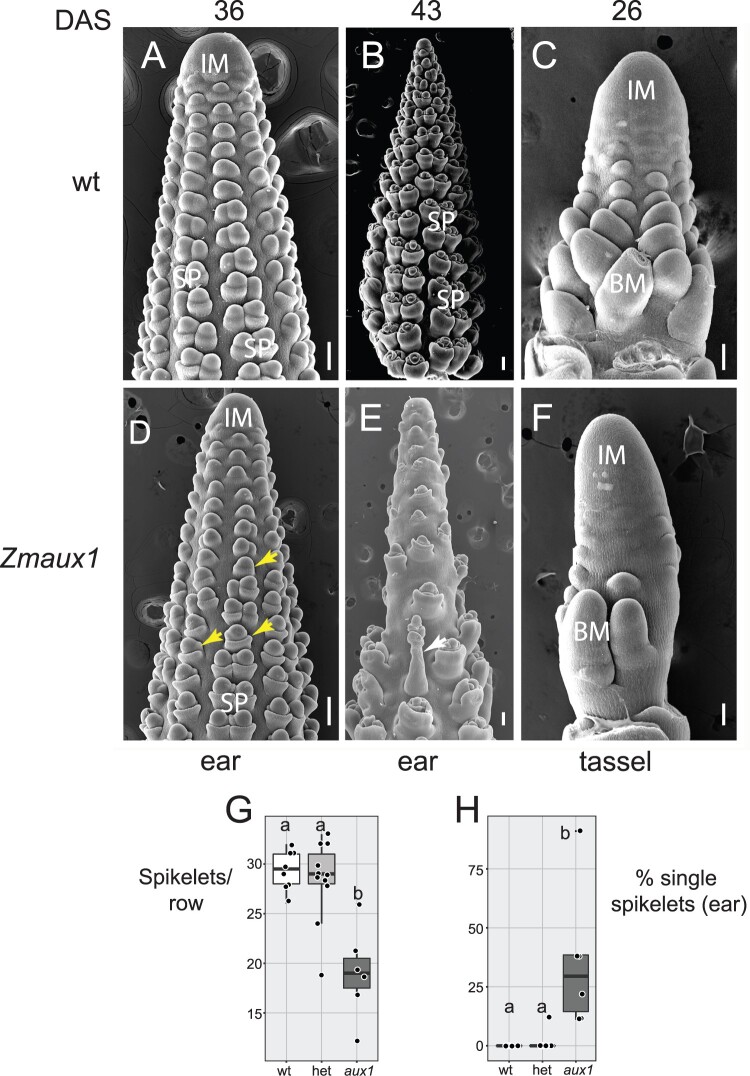
Early ear and tassel inflorescences of *zmaux1*. A–F, SEM images of WT (W22) (A–C) and *zmaux1* (D–F) inflorescences. A and B, are heterozygous WT; *Zmaux1* (C) is homozygous WT. Ears (A, B, D, and E) at 36 (A and D) and 43 DAS (B and E). Tassel at 26 DAS (C and F). Yellow arrows, single spikelets. White arrow, elongated spikelet. G, Number of spikelets per vertical row in the ear in WT (white), heterozygote (light gray) and *zmaux1* (dark gray) plants. H, Percentage single spikelets in ear. Colors as in (G). Box plots as in Figure 1. Significance values determined by ANOVA and Tukey’s HSD test. Boxes with the same letter are not significantly different at *P* < 0.05. Mean, sd, sample sizes, and *P*-values in Supplemental Table S3. Scale = 100 μm. SP, spikelet pair.

Because *S. viridis* lacks a set of auxin-related mutants, we used maize to test genetic interactions of AUX1 with other loci. The mutant for the *SPP1* ortholog in maize, *zmaux1*, produced fewer branches in the tassel and fewer spikelets per row in the ear and tassel compared to the WT (W22 inbred) and heterozygous controls (Supplemental Table S3), a phenotype analogous to that in *S. viridis* (Figure 4, A–G). Also like *S. viridis*, the mutation had no obvious effect on IM sizes (Figure 4, A–F). Spikelets in *Zea* occur in pairs, with a pair generally interpreted as a short lateral branch (Vollbrecht et al., 2005; Whipple, 2017). Therefore, if *zmaux1* affects higher order branches in the inflorescence, it should affect whether both members of the pair initiate and indeed *zmaux1* showed more single and fewer paired spikelets in both ear and tassel (Figure 4, A–F and H; Supplemental Table S3). The tips of *zmaux1* ears were often elongated as were some individual spikelets themselves (Figure 4E), similar to the spikelet-tipped bristles in the *spp1* mutant. Thus *SPP1* controls branch initiation, elongation and fate determination, but not IM size, in both *S. viridis* and maize.

**Figure 5:**
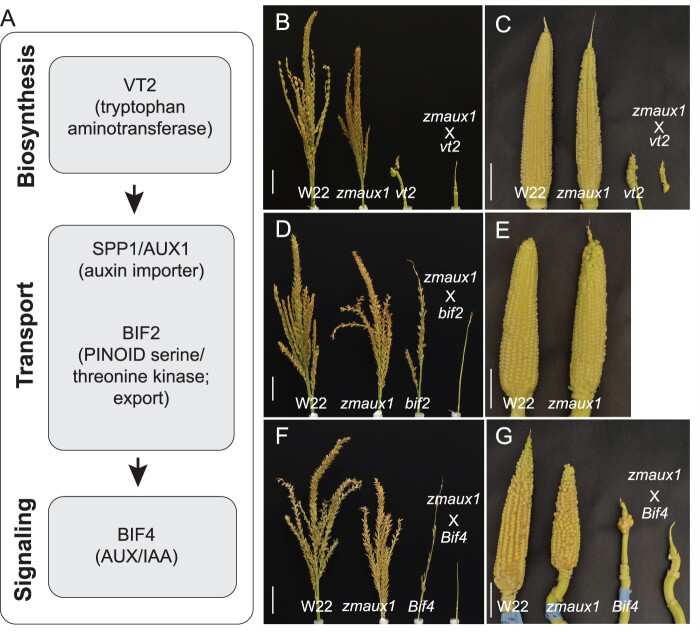
Auxin double mutant analysis in maize. A, Model showing hypothesized relationship of classic genes involved in auxin biosynthesis, transport and signaling, based on information from the literature regarding function. (B, D, and F) tassels and (C, E, and G) ears from F2 progeny of crosses between *zmaux1* and *vt2* (B and C), *bif2* (D and E), and *Bif4* (F and G). Genotypes in each panel are, left to right, WT, *zmaux1*, classical mutant, and double mutant. Most *bif2* and *zmaux1bif2* mutants fail to produce ears. Scale = 5 cm.

We crossed three well-characterized auxin mutants in maize to *zmaux1*, guided by the presumed pathway shown in Figure 5A based on their biochemical functions. These included an auxin biosynthesis mutant (*vanishing tassel 2* [*vt2*], encoding a grass-specific tryptophan aminotransferase; Phillips et al., 2011), a regulator of auxin efflux (*barren inflorescence 2* [*bif2*], encoding a serine/threonine kinase co-orthologous to PINOID in Arabidopsis; McSteen et al., 2007; Pressoir et al., 2009), and an auxin signaling protein (*Bif4*, encoding an AUXIN/INDOLE-3-ACETIC ACID (Aux/IAA) protein; Galli et al., 2015).

Plants with the mutant allele *zmaux1* had reduced branching in the ear and tassel in all three mutant families (*vt2, bif2*, and *Bif4*) (Figure 5, B–G; Supplemental Figures S3–S5). Kernel number, reflecting the total number of spikelets and hence the total number of branches, was also significantly reduced by the *zmaux1* single mutant in *vt2* and *Bif4* mutant families (*bif2* mutants failed to initiate ears), although traits that might contribute to total kernels (ear row number, spikelets per row) were not significantly lower in all cases, probably due to small sample size (Supplemental Table S3; Supplemental Figures S3, D, S4, D, and S5, D). Number of tassel branches was also significantly lower in all cases, but the number and density of spikelets on the main spike of the tassel was not always affected. In contrast, tassel length, height of the flag leaf, and total number of leaves was not significantly different for *zmaux1* mutants (Supplemental Table S3; Supplemental Figures S3–S5).

The effect of the double mutants on inflorescence characteristics is consistent with what we know about the function of the underlying genes. The locus defective in auxin biosynthesis, *vt2*, almost completely abolished branching in both the tassel and ear and suppressed growth of the tassel, thereby completely obviating any effect of *zmaux1*. *vt2* single mutants were indistinguishable from *zmaux1;vt2* double mutants for these traits (Figure 5B; Supplemental Figure S3, B–G). Likewise, BIF2 phosphorylates the auxin efflux carrier ZmPIN1 and its mutation blocks inflorescence branching, presumably by preventing auxin efflux (Skirpan et al., 2009). *bif2* single mutants were also indistinguishable from the *zmaux1;bif2* double mutant for the same branching traits as *vt2* (Supplemental Figure S4, B–G). *Bif4* encodes a protein involved in auxin signaling and creates a less severe defect in branching than *vt2* and *bif2* (Supplemental Figure S5). The *Bif4* mutant phenotype is significantly enhanced in the *zmaux1;Bif4* double mutant for kernel number, tassel branch number, and density of spikelets on the main spike of the tassel, although the effect on ear row number, spikelets per row, and spikelets on the main spike was nonsignificant (Supplemental Figure S5, B–G).

The double mutants had an unexpected effect on vegetative characteristics. As reported previously, the *vt2* and *bif2* mutations led to slight but nonsignificant reductions in flag leaf height and significant reductions in leaf number (McSteen et al., 2007; Phillips et al., 2011), an effect that was enhanced by *zmaux1*; the phenotypes of *zmaux1;vt2* and *zmaux1;bif2* were significantly more severe than either single mutant (Supplemental Figures S3, I and J and S4, I and J). Vegetative traits in the *Bif4* family were less striking than in the other families. Neither *zmaux1* nor *Bif4* single mutants significantly affected leaf number or plant height, but height to the flag leaf was significantly lower in double mutants (Supplemental Figure S5, I and J). The synergistic effect in double mutants involving all three auxin-related genes indicates that *zmaux1* does indeed function in the auxin pathway, and moreover, that auxin import has a role in normal leaf production.

### SPP1 localizes to epidermal cells in BMs in the inflorescence

SPP1 was localized in the *S. viridis* inflorescence using a translational fusion with a green fluorescent protein (GFP) fused to SPP1 (SPP1-iGFP) in an internal facing (cytoplasmic) N-terminal hydrophilic loop of SPP1 (Supplemental Figure S6A). We initially placed *SPP1-iGFP* under a constitutive promoter (*proPvUBI1::SPP1-iGFP*) to check its integrity with transient expression assays in leaves of *Nicotiana benthamiana*. SPP1-iGFP localized preferentially to a thin line at the periphery of epidermal cells, consistent with PM localization (Supplemental Figure S6, B–D).

SPP1-iGFP localization is consistent with its presumed routing through the secretory pathway to the PM as well as the nuclear membrane. Using tissue culture transformation, we introduced our *SPP1-iGFP* construct driven by its native promoter (*proSPP1::SPP1-iGFP*) to *spp1* mutants, validated three independent events by PCR genotyping, and selected one containing an expressed transgene (*spp1_T*) for further characterization (Supplemental Figure S7, A and B). SPP1-iGFP partially rescued defects in *spp1* inflorescences (Supplemental Figure S7, C–I; Supplemental Table S4). For all traits examined, the mean value for the transgenic plants was shifted in the direction of the WT value although the difference between transgenic SPP1-iGFP and *spp1_NT* was not always significant. For plant height at 34 DAS (Supplemental Figure S7D), panicle length (S7F), and spikelets per primary branch (S7H), values for SPP1∼GFP plants were neither significantly different from WT nor from *spp1_NT*, although WT and *spp1_NT* differed significantly from each other. SPP1-iGFP also significantly reduced the agravitropic root phenotype of *spp1* (Supplemental Figure S7, J and K). SPP1-iGFP thus appears to function as a weak mutant allele of SPP1 but is less severe than the original mutation.

**Figure 6:**
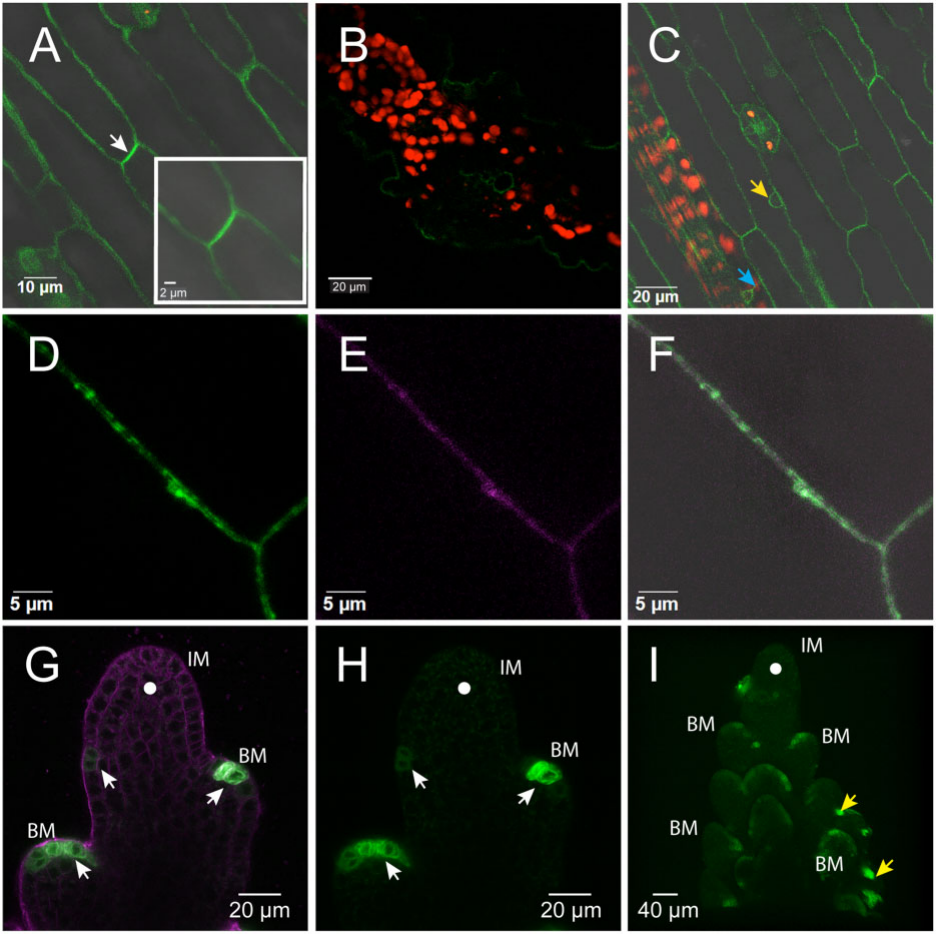
Expression pattern and subcellular localization of SPP1-iGFP in *S. viridis*. A–F, Localization of SPP1-iGFP in stably transformed *S. viridis* leaves at 8 DAS. A, Leaf surface showing fluorescent signals on the PM. Strongest signals on the PM may indicate weak polar localization (white arrowhead). B, Leaf cross section showing SPP1 expression in epidermis and veins. C, Leaf showing weak GFP signals on the transcellular strands (cyan arrowhead) extending from nucleus to PM, and around the nuclear membrane (yellow arrowhead). Red, chlorophyll autofluorescence. D–F, Leaf cells expressing SPP1-iGFP (D; green), counterstained with FM4–64 (E; magenta), visible as a thin line on the PM. Overlay (F) merges (D) and (E). (A, C–F) are single confocal sections; (B) is a projection of several sections. Scales as noted on images. G–I, Localization of SPP1-iGFP in stably transformed *Setaria* inflorescences at 11 DAS. G and H, Expression of SPP1-iGFP fusion protein in primary BMs along inflorescence flanks (white arrowheads). IM lacks fluorescent signals (closed white circle). G, Merged image of green (GFP signals) and magenta (FM4–64 signals) channels. H, Green channel only. See also Supplemental Movie S1. I, A single epidermal confocal focal plane from Supplemental Movie S2 showing epidermal enrichment of SPP1-iGFP expression in meristems of elongating primary branches. A few secondary branches also express SPP1-iGFP (yellow arrowheads). Merged image of green (GFP signals) and magenta (for FM4–64 signals) channels. For (G and H) only, green channel with 0.7 gamma correction to make dim signal on left side of meristem more apparent. All other images uncorrected and linear.

Confocal imaging in the T_3_ generation showed that in the developing inflorescence, emerging leaves, and roots, GFP signals were mostly on the cell periphery of outer epidermal layers (Figure 6, A–F; Supplemental Figure S6, E–J). SPP1-iGFP in leaves colocalized with FM4–64, a marker of the PM, confirming that the peripheral location of the signal indeed came from the membrane (Figure 6, D–F). SPP1-iGFP was also visible in a fine perinuclear line, likely to be the nuclear membrane (Figure 6C; Supplemental Figure S6, H and I), and in transcellular strands extending from the nucleus to the PM (Figure 6C).

SPP1-iGFP appeared in narrow arc at the apex of and possibly adaxial to primary BMs of the inflorescence (Figure 6, G–I; Supplemental Movie S1) but was absent from the IM itself. Expression remained undetectable in older IMs and decreased in older BMs but was visible in secondary meristems in a position analogous to that seen in primary BMs (Figure 6H; Supplemental Movie S2). Expression decreased or disappeared in older IMs and older BMs (Figure 6I; Supplemental Movie S2). SPP1-iGFP expression was consistently absent in apical regions of both IM and vegetative shoot apical meristems (Figure 6; Supplemental Movies S1–S3).

### *SPP1* affects expression of inflorescence developmental genes

We used RNA-seq to compare gene expression in A10.1 and *spp1* inflorescences at 10, 12, and 14 DAS (see Figure 3; Supplemental Tables S5–S7); transcripts were clustered with WGCNA (Langfelder and Horvath, 2008). Among the 10,434 transcripts in the analysis, we identified seven co-expression modules in A10.1 inflorescences and ten in *spp1* (Supplemental Figure S8). None of the modules was genotype specific and most were strongly preserved between genotypes (Supplemental Figure S9). For example, the largest module in A10.1 (turquoise) included 6650 transcripts with low expression at 10 DAS, moderate at 12, and high expression at 14 DAS; 5,571 of these transcripts fell into either the turquoise or blue modules in *spp1*, which showed a similar overall pattern (Supplemental Figures S8 and S9B). Most gene ontology (GO) terms were comparable between the two genotypes, but the terms “cellular response to auxin stimulus,” “response to gibberellin,” and “regulation of abscisic acid-activated signaling pathway” showed differential enrichment (Supplemental Figure S10).

**Figure 7:**
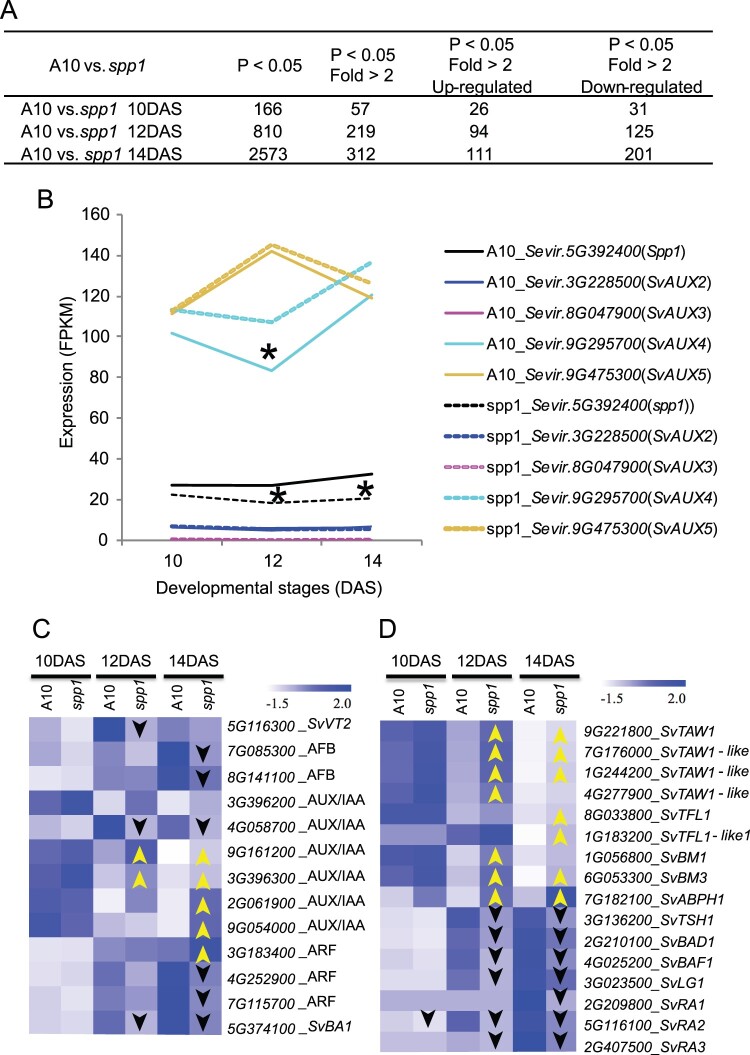
Differentially expressed genes in *spp1* inflorescences at 10, 12, and 14 DAS. A, Numbers of genes that are differentially expressed, upregulated or downregulated between WT (A10.1) and *spp1* at each time point. B, Expression of the five auxin influx carrier genes in *S. viridis* in WT and *spp1* inflorescences. C, Heat map comparing expression of selected auxin pathway-related genes in WT (A10.1) and *spp1* inflorescences. D, Heat map of selected differentially expressed genes involved in inflorescence branching. Yellow upward pointing arrows and black downward pointing arrows indicate upregulation and downregulation, respectively, compared to A10.1 at the same developmental stage. Asterisk indicates expression significantly different between mutant and WT plants at q < 0.05. Data from RNA-seq experiments, three to four biological replicates at each time point.

Consistent with the high conservation of the WGCNA expression modules, relatively few transcripts were differentially expressed between A10.1 and *spp1*. At 10 DAS, before the mutant phenotype was visible, only 166 genes were differentially expressed, 57 of which differed more than two-fold (Figure 7A; Supplemental Table S7). At 12 and 14 DAS, still only a few hundred genes were differentially expressed (Figure 7A; Supplemental Table S7), with slightly more downregulated than upregulated in the mutant compared to WT.

We investigated the expression of *SPP1* and its four homologs, *SvAUX2–SvAUX5* (Figure 7B); *SvAUX1* is *SPP1* and will be referred to as such here. *SPP1* expression in *spp1* mutants was significantly reduced at 12 and 14 DAS compared to that in A10.1 (Figure 7B), as shown previously with RT-qPCR (Huang et al., 2017). At all three time points of both genotypes, expression of *SvAUX2* was several fold lower than that of *SPP1* and *SvAUX3* was scarcely expressed at all (fragments per kilobase of transcript per million [FPKM] values <1 for all samples; Supplemental Table S6). *SvAUX4* and *SvAUX5* were more highly expressed than *SPP1* over all three time points. Among *SvAUX2–SvAUX5*, only *SvAUX4* differed significantly in *spp1* mutants, with higher expression in mutant plants than in A10.1 (Figure 7B), possibly indicating a compensation effect. *SPP1*, *SvAUX2*, *4*, and *5* belong to the turquoise module in A10.1, members of which are downregulated at 10 DAS but upregulated by 14 DAS. In *spp1* mutants, the expression pattern reverses for *SPP1* and *SvAUX2* (Supplemental Figure S11).

Only a few auxin-related genes differed significantly in expression between genotypes (Figure 7C; Supplemental Table S8). In A10.1 these fell into the turquoise, blue and brown modules (Supplemental Figure S11), which together include most of the transcripts. *SvVT2* and two auxin signaling F-box binding genes (encoding potential auxin receptors), and a homolog of *BARREN STALK1/LAX PANICLE 1 (SvBA1*, encoding a basic helix*–*loop*–*helix protein expressed downstream of auxin signaling; Komatsu et al., 2003; Gallavotti et al., 2004; Galli et al., 2015) were downregulated at 12 and/or 14 DAS (Figure 7C). While five of the six *AUX*/IAA genes were upregulated in the mutant, one (*4G058700*_AUX/IAA) was downregulated (Figure 7C; Supplemental Table S8).

Genes whose homologs in maize are important for branch initiation and boundary formation were all downregulated in *spp1* (Figure 7D; Supplemental Table S7), including homologs of *TASSEL SHEATH1* (*TSH1*, encoding a GATA transcription factor [TF]; Wang et al., 2009; Whipple et al., 2010), *BRANCH ANGLE DEFECTIVE1* (a TCP TF; Bai et al., 2012), *BARREN STALK FASTIGIATE1* (an AT-hook protein; Gallavotti et al., 2011) and *LIGULELESS 1* (a nuclear localized protein; Moreno et al., 1997; Lewis et al., 2014). Expression of homologs of the RAMOSA pathway genes *RA1* (encoding a Cys2-His2 zinc-finger TF; Vollbrecht et al., 2005), *RA2* (a *LATERAL ORGAN BOUNDARY* domain TF; Moreno et al., 1997; Bortiri et al., 2006) and *RA3* (encoding a trehalose-phosphate phosphatase) (Satoh-Nagasawa et al., 2006) was also lower in *spp1* (Figure 7D; Supplemental Table S7). Expression levels are standardized to reflect relative, rather than absolute, expression, so the downregulation is unlikely to reflect the lower number of branches in *spp1*. However, all the branching genes are downstream in pathways that are ultimately regulated by auxin and auxin transport, as seen in mutations of other transport-related proteins such as Bif2 (Skirpan et al., 2009: Gallavotti, 2013).

In contrast, homologs of genes promoting IM identity and increased production of primary branches were upregulated (Figure 7D; Supplemental Table S7), including *TAWAWA1* (*TAW1*; Yoshida et al., 2013) and *TERMINAL FLOWER1* (*TFL1*; Nakagawa et al., 2002; Danilevskaya et al., 2010; Hanano and Goto, 2011). A homolog of *ABERRANT PHYLLOTAXY 1* (*ABPH1*; a cytokinin-inducible type A response regulator), which controls phyllotactic patterning and meristem size (Lee et al., 2009), was also significantly upregulated in *spp1* (Figure 7D; Supplemental Table S7), as were homologs of *BROWN MIDRIB 1* and *3* (*SvBM1* and *SvBM3*) (Figure 7D; Supplemental Table S7). While BM1 and BM3 are involved in lignin synthesis in maize (Vignols et al., 1995; Halpin et al., 1998), they also affect kernel number, plant height, and days to flowering (Pedersen et al., 2005), traits associated with *spp1/aux1* mutations.

### SPP1/SvAUX1, but not the other four AUX1 homologs, is necessary for inflorescence branching

We used CRISPR–Cas9 technology with two guide RNAs to introduce mutations into all five putative auxin importers in accession ME034V, used for its high transformation efficiency (Zhu et al., 2017; Supplemental Figure S12A). We obtained two independently edited single mutants in *svaux1*; we call these *spp1-C* for *spp1-CRISPR*. *spp1-C* exhibited a phenotype similar to that of the *spp1* mutant in the A10.1 background (Supplemental Figure S12B). We also retrieved two double mutants, *spp1-C*, *svaux5* (*spp1-C*,*aux5*), and *spp1-C svaux3* (*spp1-C*,*aux3*), one triple mutant, *spp1-C*, *svaux2svaux5* (*spp1-C*,*aux2,5*) and two quintuple mutants, *spp1-C, svaux2svaux3svaux4svaux5* (*spp1-C, aux1,2,3,4,5*) (Supplemental Figure S12B). One quintuple mutant, line cz66-11-16-11-1-4, had edits in all five homologs, with indels in *SvAUX2–SvAUX5* likely to knockout gene function because of frameshifts. However the *spp1-C* edit in *SvAUX1* resulted in a single nonsynonymous substitution (Supplemental Figure S12B), substituting an aliphatic residue (leucine) for an aromatic one (phenylalanine) in a presumed transmembrane domain (Supplemental Figure S6A); both residues are hydrophobic and will have limited effect on charge. We inferred that *Spp1-C* in this line could still be functional, leaving the line with only four mutated SPP1 homologs. Here we refer to this line as *svaux2svaux3svaux4svaux5* (*aux2,3,4,5*).

**Figure 8:**
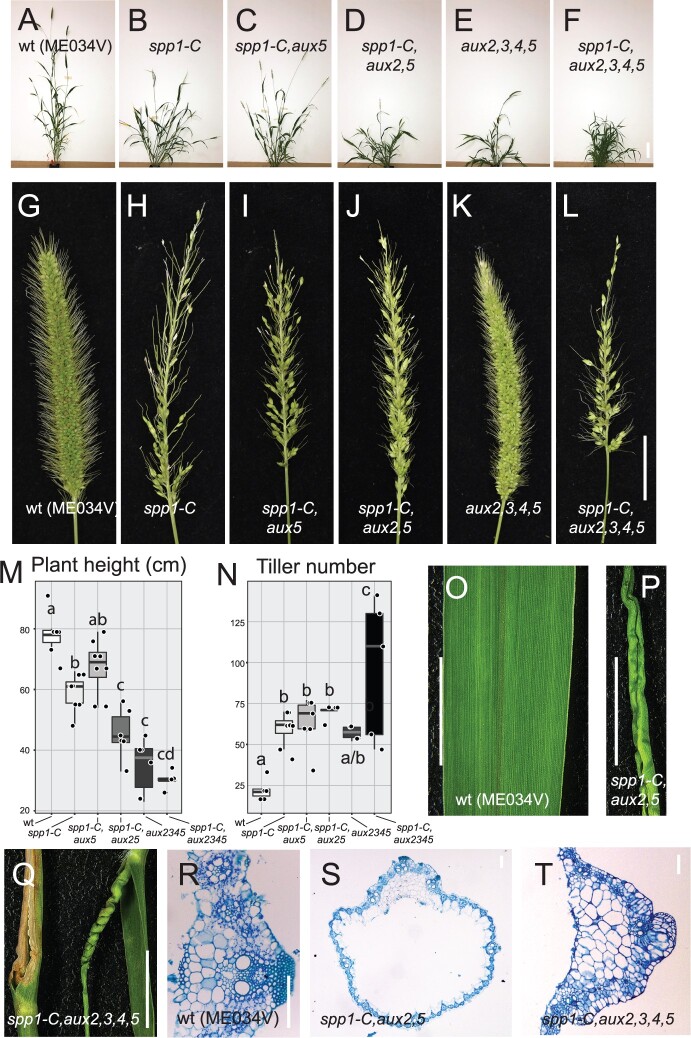
Auxin importer gene mutants in *S. viridis*. A–F, WT and mutant plants photographed at 58 DAS, showing relative height and extent of tillering. A, WT (ME034V); (B) *spp1-C*; (C) *spp1-C,aux5*; (D) *spp1-C,aux2,5*; (E) *aux2,3,4,5*; (F) *spp1-C,aux 2,3,4,5*. *aux 1,3* not available for this set of photos. Scale = 10 cm. G–L, WT and mutant inflorescences from the same plants and on the same day as in (A–F). Scale = 2 cm. M and N, Plant height (cm) (M) and number of tillers (N) on each plant 10 weeks after sowing. Box plots as in Figure 1. Fill colors white (WT; ME034V) to black (*spp1-C,aux 2,3,4*,5), with different gray values for different numbers of mutant loci. Significance values determined by ANOVA and Tukey’s HSD test. Boxes with the same letter are not significantly different at *P* < 0.05. Mean, sd, sample sizes, and *P*-values in Supplemental Table S9. O, WT ME034V leaf. Scale bar = 1 cm. P and Q, Leaves in *spp1-C,aux2,5* or *spp1-C,aux 2,3,4*,5 mutants showing tube shape (P, right leaf in (Q)), early senescence in the tips (left leaf in (Q)), and twisted shape (right leaf in (Q)). Scale bar = 1 cm. R–T, Leaf cross sections from WT (R), *spp1-C,aux2,5* (S) and *spp1-C,aux 2,3,4*,5 (T) mutants. Toluidine blue staining. Scale bar = 100 μm.

All *SvAUX* mutants except *spp1-C,aux5* were significantly shorter than WT at 10 weeks, although leaf number was not significantly affected (Figure 8, A–F and M; Supplemental Table S9). Tiller number in WT plants did not differ between 4 and 10 weeks of growth, and the mutants did not differ amongst themselves at either stage (Figure 8, A–F and N; Supplemental Table S9). However, tiller number in the mutants was significantly higher than WT at 10 weeks. Because *aux2,3,4,5* had more tillers, one of its the four mutant AUX loci likely contributes to the tillering phenotype in addition to *spp1-C* (Figure 8, A–F and N).

Inflorescences of higher order mutants involving *spp1-C* were similar to those of *spp1-C* single mutants (Figure 8, G–L), supporting our hypothesis that SPP1 is the major auxin influx carrier regulating inflorescence branching. Conversely, inflorescences of *aux2,3,4,5* were morphologically similar to those of WT (Figure 8, G and K), implying that the F377L substitution in SPP1 in that line indeed does not affect its function and that SPP1 alone is sufficient for inflorescence branch formation. Panicle length did not vary significantly among the plants, except that the panicle of *aux2,3,4,5* was slightly shorter, a difference that was just barely significant (Supplemental Table S9).

The higher order mutants also exhibited phenotypes not observed in WT or single mutants (*spp1-C* or *spp1*). For example, *spp1-C,aux2,5*, and *spp1-C,aux2,3,4,5* often produced twisted or tube-shaped leaves, or leaves that senesced prematurely with yellowing tips and edges (Figure 8, O–Q). The vasculature in the abnormal leaves was mis-patterned, with abnormalities in midrib cell layers and organization (Figure 8, R–T). Leaves in the upper part of the plant (those initiated latest), particularly the flag leaves, were affected more noticeably than those that initiated earlier. Lateral root number in *spp1-C,aux2*,5, and *spp1-C,aux2,5* was also reduced but primary root length was unaffected (Supplemental Figure S12, C–E).

## Discussion

The effect of SPP1 mutations on the inflorescence in *S. viridis* (*spp1/SvAUX1*) is strong, easily observed, and not obscured by mutations in its four paralogs, unlike mutations in AUX1 orthologs and paralogs in other species such as Arabidopsis. The clear mutant phenotype has allowed us to uncover and validate numerous developmental roles for the auxin importer, including several that had not been observed in other systems. We were specifically interested in the role of SPP1 in inflorescence branching but also identified functions in stigma branching, formation of higher order inflorescence branches, and glumes (leaf-like floral bracts) which together affect plant fertility (yield).

Of the five AUX1-like proteins in *S. viridis*, SPP1/SvAUX1 has the major effect on inflorescence branching, although we cannot fully rule out the possibility that the other homologs could have a weak effect on their own. Consistent with this, AUX2 and AUX3 have low to no expression during inflorescence development. AUX4 and AUX5 are highly expressed during early inflorescence development, but mutations in these genes do not further enhance the sparse panicle phenotype of *spp1*; instead they lead to shorter plants. Assuming that the model of auxin flow in *S. viridis* is similar to that demonstrated in other species (e.g. O’Connor et al. 2014), we speculate that AUX4 and AUX5 proteins could participate in internal basipetal auxin transport from auxin maxima at the branch initiation sites, whereas SPP1/AUX1 is likely mediating auxin movement to the branch initiation sites in the outer cell layers. Future imaging of the localization and dynamics of these auxin influx carriers is necessary to test this hypothesis.

Although SvAUX2–SvAUX5 make minimal or no contribution to inflorescence branching, they are collectively important for plant height, tiller formation, and leaf development. Reduced plant height and increased tiller number, as seen in higher order mutants, indicates a loss in apical dominance, a characteristic function of auxin. Twisted leaves are also seen in maize mutants whose auxin function is compromised, such as *growth regulating factor-interacting factor1* (Zhang et al., 2018) and *rough sheath2* (Tsiantis et al., 1999).

### SPP1 regulates multiple aspects of inflorescence development downstream of meristem maintenance

The *spp1* mutant has fewer primary inflorescence branches, fewer higher order inflorescence branches, an altered ratio of bristles to spikelets, and defective stigmas, indicating that SPP1 controls branch initiation and elongation and meristem fate determination. The *zmaux1* mutant was also abnormal in these aspects, suggesting the role of SPP1 is likely conserved in the panicoid grasses. However, IM size is not affected in *spp1*, suggesting that SPP1 controls inflorescence development independent of meristem maintenance in grasses. This is consistent with findings from Arabidopsis, where the quadruple mutant of *aux1lax1lax2lax3* had a normal meristem, despite its defects in phyllotactic patterning (Bainbridge et al., 2008).

The only defective floral organ in *spp1* is the gynoecium, whereas other auxin-related grass mutants, such as *ba1* (Gallavotti et al., 2004) and *bif2* (McSteen and Hake, 2001), aborted multiple floral organs. Stigmas in most grasses are highly branched, and our data suggest that auxin transport is necessary for appropriate branch formation. We also find that *spp1* mutants have fewer styles and stigmas on approximately half of the florets in any given plant, but the effects are more continuous and quantitative than shown in Figure 2, D–H. In addition, position of the spikelet in the inflorescence and along the branch may affect gynoecial development. Mutations in other genes such as those of a SHORT INTERNODES family TF (Yuo et al., 2012) also affect stigma morphology, suggesting that a specific network of genes regulating stigma formation remains to be discovered. The stigma defects could contribute to reduced fertility in *spp1*, although auxin is also involved in fertilization and seed development (Robert et al., 2015; Figueiredo and Köhler, 2018), which were not investigated here. The *spp1* mutant and tagged line may provide tools for a deeper investigation of the role of auxin transport in gynoecial patterning and function.

### SPP1 affects regulation of many branching-related genes, but not wholesale rewiring of the transcriptome

The number of genes affected by disrupting *spp1* is not large but does include loci known to control inflorescence architecture in other systems. Downregulated genes (Figure 7B) in *spp1* mutants contribute to production of fewer primary and higher order branches. In maize, the adaxial boundary of the axillary meristem is established by BA1, which is expressed very early in inflorescence development and is required for branch production (Gallavotti et al., 2004). The subtending bract is also required and needs to be suppressed for normal development, a process controlled by TSH1 (Chuck et al., 2010; Whipple et al., 2010). The meristem is delimited by RA2, which along with RA3 regulates RA1 (Eveland et al., 2014). Antagonism between the RA signaling network and the TSH network specifies which cells are allocated to the bract versus the meristem (Whipple et al., 2010; Xiao et al., 2021). All these gene networks—BA1, TSH1, and RA1/2/3—are affected if auxin synthesis, transport, or signaling are disrupted (Gallavotti et al., 2008b, Gallavotti, 2013). However, we note that TSH4 (a Squamosa promoter-binding protein TF) (Chuck et al., 2010; Whipple et al., 2010) is upstream of TSH1 (Xiao et al., 2021). Expression of *TSH4* is unchanged in *spp1*.

While the downregulated genes may explain the limited branching in *spp1*, the upregulated genes could help explain the paucity of spikelets. These genes include putative orthologs of *TAW1* and *TFL1/CENTRORADIALIS* both of which lead to more branching and fewer spikelets when over-expressed in rice (Nakagawa et al., 2002; Kyozuka, 2014). SvBM1 and SvBM3 are putative orthologs of the corresponding maize and sorghum *brown midrib* genes, which control lignin production but also affect flowering and grain yield by unknown mechanisms (Pederson et al., 2005). The effect of over-expression of ABPHYL1, a cytokinin-inducible response regulator, is unclear. When ABPHYL1 is mutated meristems become larger (Giulini et al., 2004), suggesting that higher gene expression would lead to smaller meristems, possibly reducing production of lateral organs. However, we did not observe a change in meristem size in *spp1* mutants.

Several (but not all) of the genes encoding AUX/IAAs and ARFs are differentially expressed in *spp1* mutants, but do not respond in a consistent manner, with some being upregulated and others downregulated. Regulation of these genes is complex and apparently tissue-specific (Galli et al., 2015; Powers and Strader, 2019), so interpretation of the expression results reported here will required more in-depth investigation.

Because SPP1 is a presumed transporter, effects on transcription must be indirect and are likely responding to levels of auxin. Even without active auxin import into the cell, it is still able to diffuse into the cell but this is (presumably) a less tightly controlled process than transport. Thus the genes and processes that are downregulated are likely to be ones that require both rapid and precisely timed active transport.

The SPP1-iGFP protein is expressed in a restricted domain at the apex of the primary BM, potentially close to the expression domain of *Ba1/Lax1* in maize and rice (Komatsu et al., 2003; Gallavotti et al., 2004). While co-localization studies would be required to verify this apparent contiguity, it is consistent with the observed downregulation of *Ba1* in *spp1* mutants. Our data also add SPP1/AUX1 to the list of auxin transporters showing epidermal localization (Kubeš et al., 2012; Balzan et al., 2014; Swarup and Bhosale, 2019).

SPP1-iGFP only partially rescued the effects of the SPP1 mutation, so our localization results need to be interpreted with caution although partial rescues are common (Stam et al., 1997). We believe that the cellular localization of SPP1-iGFP, largely in the cell membrane and in perinuclear strands surrounding the nucleus, is likely to be accurate. We did not observe (or expect) cytoplasmic localization. However, even with proper localization, protein function may be impaired if the tag interferes with posttranslational modifications, protein turnover, position within the membrane, interactions with other proteins, or interactions with auxin. We hypothesize that SPP1-iGFP may import less auxin into the cells, creating a weak allele that helps rescue some effects of the mutation but is not fully functional.

### Function of SPP1 in Setaria applies also to maize and possibly other panicoid grasses

We extended our observations to maize, where, by manipulating other aspects of auxin synthesis, transport and signaling, we showed that ZmAUX1 shares developmental functions with SPP1. We also confirmed that it functions as expected in combination with known auxin-related mutants.

ZmAUX1 also influences leaf number, a defect that has been shown previously in other auxin-related mutants such as *vt2* (Phillips et al., 2011), in which adult leaves are missing, *Hoja loca* (Richardson et al., 2021), in which some leaves fail to initiate, as well as *bif2* (McSteen and Hake, 2001), *sparse inflorescence1* (Gallavotti et al., 2008a) and *Bif1* (Barazesh and McSteen, 2008). The nature of the leaf production defect for *zmaux1* is unknown. In contrast, mutations in *SPP1* or the other *SvAUX* loci, did not significantly affect leaf number (Supplemental Table S9), although the developmental stages may not have been comparable between the two species.

In summary, we suggest that the *spp1*, *spp1-C* mutants and the SPP1-iGFP tagged line could provide useful tools with which to develop broader models of auxin flux into and out of cells. While most of the phenotypes we report are not unexpected for a protein that affects auxin, they show that auxin influx exerts a more extensive control over plant development than previously known. In particular, SPP1/SvAUX1 is clearly a central player in the genetic network that modulates all above-ground branching and could be used to test models of auxin regulation. Whether the effects we see in Setaria and maize indicate a fundamental difference between monocots and dicots in the role of auxin influx awaits testing in a broader set of species.

## Materials and methods

### Plant growth, phenotyping, and statistical comparisons

Green millet (*S.* *viridis*) accessions A10.1 and ME034V were grown in growth chamber and greenhouse conditions, respectively, following Acharya et al. (2017) and Zhu et al. (2018). The original *spp1* mutation was isolated from an A10.1 background; ME034V was chosen for CRISPR confirmation of the mutant phenotype because of its high transformation efficiency. Plant height, leaf number, panicle length, and branch number were measured as described in Huang et al. (2017) and Zhu et al. (2018). Fertility was measured as the ratio of spikelets with a fully developed upper floret to total spikelets; bristles were ignored for fertility measurements. Tillers were counted at 37 DAS and plant height measured at 40 DAS. Stigma and style number were assessed by dissecting one floret from each of 10 spikelets per plant, five plants for *spp1* and three for A10, for a total of 50 mutant florets and 30 WT. We recorded number of styles and number of stigmas for each floret. Numbers varied as much within as between plants so the numbers were pooled among all plants to estimate the frequency of each number. Histology and SEM followed Zhu et al. (2018). Inflorescence length, meristem width and height were measured using ImageJ (Schneider et al., 2012) from SEM photos.

For root phenotyping, sterilized seeds were grown either in Murashige and Skoog (MS) medium or germination pouches as described in Huang et al. (2017) and Acharya et al. (2017), respectively.

Auxin rescue experiments followed Marchant (1999) and Yu et al. (2015). 2, 4-D (from PlantMedia (Dublin, OH, USA) in 1-mM stock with pure ethanol) and NAA (from Sigma-Aldrich, St Louis, MO, USA), in 10-mM stock with pure ethanol) were added to the medium to a final concentration of 0.1 mM. MS medium containing 0.1% ethanol (v/v) was used as a mock control. Seeds were grown on MS medium for 3 d and then transferred to media containing appropriate concentrations of auxin or mock for 3 more days. Root hairs were imaged at 4× magnification on a Leica DM750 microscope. Root hair number was counted in the focal plane on the side of the root facing the observer and normalized to root length. Experiments were repeated 3 times.

In maize, *zmaux1* mutant plants were crossed to *vt2*, *bif2* and *Bif4* mutants, and F2 segregating populations were grown in the field in Columbia, Missouri in 2017. Plants were genotyped to identify single and double mutants using primers listed in Supplemental Table S10 and were phenotyped at the eighth week. For the dominant mutant *Bif4*, both heterozygotes and homozygotes were included for mutant phenotyping analysis. For each mutant and mutant combination, we assessed traits of the tassel (length from flag leaf to tassel tip, number of branches, spikelets on main spike, and spikelet number per cm) and ear (kernel number and ear row number), and three vegetative traits (height of flag leaf, number of leaves above the lowest elongated internode, and number of tillers).

All pairwise comparisons used Welch’s *t* test as implemented in R (R Core Team, 2020). Single, double and higher order mutants were compared to each other and to WT by one-way or two-way Type I or II ANOVA as appropriate, followed by Tukey’s honestly significant difference test using standard programs in R (R Core Team, 2020). Comparisons with *P* > 0.05 were considered nonsignificant.

### Generation of mutants for auxin importer gene homologs

Cloning of CRISPR–Cas9 constructs and *S. viridi*s transformation followed Zhu et al. (2021). Two guide RNAs targeting GGGAGATCATGCACGCGATG and AGTTGATGGGCCCGAAGAAG, respectively, were designed to target all five *SvAUX1* paralogs and used in the CRISPR-Cas9 constructs. Briefly, we used the module 2 vector pMOD_B2518 via the Esp3I/BsmBI cloning site for the first guide RNA and the module 3 vector pMOD_C2616 via the BsaI cloning site for the second. Then, the AarI cloning site in the destination vector pTRANS_250d was used to assemble the module 2 and 3 vectors plus the module 1 vector pMOD_A1110 containing Cas9 and hygromycin phosphotransferase genes. Constructs were sequenced to verify integrity. The assembled vector was transformed into the Agrobacterium strain AGL1, which was then transformed into the accession ME034V. More than ten transgenic plants were obtained and gene edits in the auxin importer genes were examined using primers listed in Supplemental Table S10. Stable homozygous lines in T3 or T4 were used for phenotypic analysis.

### RNAseq sampling, sequencing, and analysis

Inflorescences from both A10.1 control plants and *spp1* mutants (in the A10.1 background) were dissected at 10, 12, and 14 DAS for RNA extraction and library preparation following Zhu et al. (2018). For each genotype and developmental stage, 10–30 inflorescences were dissected and pooled to constitute a biological replicate; the exact number depended on the size of the inflorescences at that stage. Three to four biological replicates were collected per genotype and stage. Specimens were collected within a 2-h window in the morning to control for circadian effects. 100-bp paired-end sequences were produced on the Illumina HiSeq 2500 platform at the University of Illinois at Urbana-Champaign W.M. Keck Center.

Adaptors and low-quality reads were trimmed using Trimmomatic (Bolger et al., 2014) and reads were quality-checked using fastqc after trimming. The *S. viridis* reference genome (version 1) was indexed using bowtie 2 (Langmead and Salzberg, 2012) from Sviridis_311_v1.0.fa.gz file at PhytozomeV11 (phytozome.jgi.doe.gov). Reads were mapped to the reference genome using tophat2 and differentially expressed genes were identified using cuffdiff (Trapnell et al., 2012). Expression levels quantified in FPKM were extracted for 35,214 *S. viridis* primary transcripts (Supplemental Tables S5 and S6). Gene annotation and grass homolog identification followed Zhu et al. (2018). Expression differences were considered statistically significant if *q* < 0.05, where *q* is a *P*-value adjusted for multiple tests that optimizes the False Discovery Rate.

Genes with an average FPKM ≥ 5 per sample group (three to four biological replicates) were extracted, and the log_2_(FPKM + 1) of genes within the top 75% of the highest median absolute deviation across three developmental stages was selected for co-expression analysis (nGenes = 10,434 from both genotypes). A co-expression network was constructed for each genotype using the R package WGCNA (version 1.70; Langfelder and Horvath, 2008) with an established pipeline (Yu et al., 2020), with blockwiseModules function and the following parameters: soft-thresholding power of 18, minModuleSize of 100, detectCutHeight of 0.995, mergeCutHeight of 0.25, deepSplit of 2. Degree distributions in each individual network followed the power law and satisfied the scale-free topology. Conservation of modules was tested with the modulePreservation function in the WGCNA package (Langfelder et al., 2011) following Yu et al. (2020). An improved *S. viridis* GO annotation was generated by the GOMAP annotation pipeline (Wimalanathan and Lawrence-Dill, 2021). A total of 33,391 of 35,214 genes (representing 94.8% of primary transcripts in the *S. viridis* genome version 1.1) were successfully annotated, with the median number of annotation terms per gene of 8. GO enrichment analysis and visualization used the R package clusterProfiler (version 4.0; Wu et al., 2021). The chord diagram of changes in module membership was plotted with R package circlize (version 0.4.13).

### Creation of SvSPP1-iGFP fusion protein, subcellular localization, and transgenics

Binary vectors were built using standard Golden Gate assembly (Werner et al., 2012). *SPP1* was internally tagged (hereafter, *SPP1-iGFP*) and placed either under the native *SvSPP1* (*proSvSPP1::SPP1-iGFP*) or a constitutive *Panicum virgatum UBI1* promoter (*proPvUBI1::SPP1-iGFP*). We were unable to transform *S. viridis* with the C-terminal fusion of GFP (SvSPP1-GFP), a problem also encountered in Arabidopsis by Swarup et al. (2004) for C- and N-terminal reporter fusions of auxin influx carriers including AtAUX1. Hence, we chose an internal facing (cytoplasmic) N-terminal hydrophilic loop of SPP1 because a similar AtAUX1 construct retained its topology and physiological role (Swarup et al., 2004). The GFP sequence in SvSPP1-iGFP was inserted between Lys_121_ and Asn_122_ (Supplemental Figure S6A), predicted to be in a hydrophilic loop (Swarup et al., 2004). 3kb of *SvSPP1* upstream sequence was PCR amplified using genomic DNA and used as *proSvSPP1*. *SPP1a* (1–363)*, SPP1b* (363–1,470) and *GFP* fragments were PCR-amplified using either cDNA or the plasmid *pL0M-C2-eGFP-15095* as templates; primers are listed in Supplemental Table S10. Each PCR fragment was cloned individually into the level 0 vectors *pICH41233* (*proSvSPP1*), *pICH41258* (*SPP1a*), *pAGM1299* (*GFP*), and *pAGM1301* (*SPP1b*). The resultant level 0 constructs plus level 0 *Nopaline synthase* terminator (NosT) vector were subsequently cloned in the level 1 vector *pICH47742* to produce *pICH47742-proSvSPP1::SvSPP1-iGFP::NosT*. The Level 1 construct *pICH47802*-*proZmUBI1::HPT*, an expression cassette with a functional *HPT* (*hygromycin phosphotransferase*) gene under a constitutive *Z.* *mays UBIQUITIN 1* promoter (*proZmUBI1::HPT*), and the *pICH47742-proSvSPP1::SvSPP1-iGFP::NosT* were then assembled in the binary level 2 vector *pICSL4723*.

The binary vector was transformed in *Agrobacterium tumefaciens* strain AGL1 for transient (*N.* *benthamiana*) or transgenic (*S. viridis*) expression analysis. To check transient expression, 6-week-old *N.* *benthamiana* leaves were agro-infiltrated following Cho et al. (2015). After 4 d, GFP fluorescence was visualized using an HC PL APO 40×/1.10 W CORR CS2 objective lens on a Leica SP8-X (Wetzlar, Germany) confocal laser scanning microscope. We used the 489 nm excitation line of the white light laser (WLL) for GFP and chlorophyll, while fluorescence emission was captured by the hybrid (HyD) detector. The detector signals were adjusted with an offset value of 0 for all channels and gain values of 90 (GFP), 10.8 (chlorophyll), and 285.4 (bright field) and with the Acousto-Optical Beam Splitter laser intensity set at 21%. Excitation and emission wavelengths for GFP and chlorophyll were 488–600 nm and 673–726 nm, respectively.

The binary vector was stably transformed into the *spp1-1* mutant line (Huang et al., 2017) at the Donald Danforth Plant Science Center Plant Transformation Facility (St Louis, MO, USA). Five putatively transgenic plants were obtained and presence of GFP was confirmed in three of them using PCR genotyping with GFP-specific primers in the T_0_ generation. One line confirmed to lack the transgene was carried forward to control for possible effects of tissue culture; this line is referred to here as *spp1_NT* (for nontransformed). One line homozygous for the transgene (GFP) was chosen and its stable expression was used in subsequent generations for confocal imaging (T_3_) and phenotypic analysis (T_4_). This line is referred to here as *spp1_T*. Primers for genotyping and expression assays are listed in Supplemental Table S10.

Relative expression was quantified for *GFP* by RT-qPCR. Four days after sowing leaves (third leaf base; *N* = 4 plants, pooled) and 11 DAS primary inflorescences (*N* = 5 plants, pooled) were hand-dissected as described in Li et al. (2010) and Huang et al. (2017), respectively. Data are the mean of three technical replicates of expression values from pooled leaves and inflorescence tissues. Total RNA was extracted using an RNeasy Plant Mini Kit (Qiagen, Hilden, Germany) and quantified using a NanoDrop 1000 spectrophotometer (Thermo-Fisher). Each RNA sample was reverse-transcribed to cDNA after DNase I treatment using a PrimeScript RT reagent kit (Takara, Shiga, Japan). PCR was performed as described in Kumar et al. (2017). Expression data for *GFP* were normalized to expression of reference genes *Sevir.2G354200* and *Sevir.9G574400* as described in Huang et al. (2017). The normalized relative quantity of GFP transgene to the two reference genes was estimated using the Comparative CT Method (ΔΔ^CT^ method) (Schmittgen and Livak, 2008).

### Image capture, analysis, and processing

Confocal images were captured on a Leica TCS SP8 confocal laser scanning microscope with an HC PL APO CS2 63×, 40×, and 20×/1.20 WATER objective lens (Leica Microsystems, Mannheim, Germany) and Leica Application Suite X (LAS X) software. The light source was the WLL for GFP, chlorophyll, and FM4–64, while emission fluorescence was captured by the hybrid (HyD) detector. Excitation and emission wavelengths for GFP, FM4–64 and chlorophyll were 430/480 nm, 490/550 nm, and 561/673–726 nm, respectively. For bright field images, a conventional photomultiplier tube (PMT) for transmittance was used (PMT trans in LAS X software). For image capture, line averages and frame accumulations were 6–16 (for roots) and 3–6 times (for inflorescence and leaves) to reduce noise. Inflorescence and shoot meristems and leaf cross sections were imaged as Z-stacks; images were reconstructed using Imaris x64, 7.2.3 (www.bitplane.com) with background subtraction settings enabled. SPP1-iGFP cellular localization in transgenic tissues was observed through multiple confocal sections. Four or five inflorescences from 11 DAS plants were dissected under the stereomicroscope and analyzed. The hand-dissected shoot apical meristems and the fourth leaf base from 6 DAS plants were embedded in 6% agarose, sectioned using a Vibratome (1500 Sectioning System), and stained using FM4–64 as described by Grandjean et al. (2004) before imaging.

All images in this article were resized as necessary, adjusted for brightness, and assembled into figures in Adobe Photoshop. Images were then imported into Adobe Illustrator for labeling. Graphs were produced with ggplot2 in R and also imported into Illustrator to adjust labels and line width.

### Data availability

Raw sequence reads for RNA-seq for the *spp1* mutant and A10.1 are deposited in the NCBI Gene Expression Omnibus (GEO) under number GSE193344. The same reads for A10.1 were also deposited previously at GEO, accession number GSE118673 (Zhu et al. (2018). Raw phenotype data are in datadryad accession number Dryad, Dataset, https://doi.org/10.5061/dryad.0zpc86701.

## Supplemental data

The following materials are available in the online version of this article.

**Supplemental Figure S1.** Additional phenotypes of *spp1* mutant plants.

**Supplemental Figure S2.** Auxin rescue experiments.

**Supplemental Figure S3.** Phenotype of *zmaux1vt2* double mutants.

**Supplemental Figure S4.** Phenotype of *zmaux1bif2* double mutants.

**Supplemental Figure S5.** Phenotype of *zmaux1Bif4* double mutants.

**Supplemental Figure S6.** Cellular localization of SPP1-iGFP.

**Supplemental Figure S7.** Validation of SPP1-iGFP in transgenic *S*. *viridis*.

**Supplemental Figure S8.** Gene co-expression modules.

**Supplemental Figure S9.** Comparisons between wild *S. viridis* A10.1 and *spp1* mutant networks.

**Supplemental Figure S10.** GO enrichment.

**Supplemental Figure S11.** Chord diagram illustrating how WGCNA module membership differs between genotypes.

**Supplemental Figure S12.** Mutants of auxin importer genes.

**Supplemental Movie S1.** Localization of SPP1-iGFP in stably transformed *Setaria* inflorescence apical meristem.

**Supplemental Movie S2.** Localization of SPP1-iGFP in stably transformed *Setaria* inflorescence apical and BMs.

**Supplemental Movie S3.** Localization of SPP1-iGFP in stably transformed Setaria vegetative shoot apical meristem.

**Supplemental Table S1.** Phenotypic comparisons between A10.1 and *spp1* mutants.

**Supplemental Table S2.** Phenotypic comparisons between A10.1 and *spp1* mutants over development.

**Supplemental Table S3.** Phenotypic comparisons between W22 (maize WT), *zmaux1* and single and double mutants of selected genes in the auxin pathway.

**Supplemental Table S4.** Phenotypic comparisons between A10.1, spp1_T and spp1_NT, testing for complementation of SPP1∼GFP.

**Supplemental Table S5.** RNA-seq library sequencing and mapping statistics.

**Supplemental Table S6.** Expression of all *S. viridis* genes from each replicate (R1–R4) of the developmental stages (10, 12, and 14 DAS) in A10.1 and *spp1*.

**Supplemental Table S7.** Expression of differentially expressed genes between A10.1 and *spp1* at each developmental stage (10, 12, and 14 DAS).

**Supplemental Table S8.** Expression of auxin pathway-related genes in A10.1 and *spp1* at each developmental stage (10, 12, and 14 DAS).

**Supplemental Table S9.** Phenotypic comparisons among AUX CRISPR mutants.

**Supplemental Table S10.** Primers used in this study.

## Supplementary Material

kiac115_Supplementary_Data

## Notes

E.A.K., C.Z., and M.S.B. designed the project. Experiments and data analysis were carried out by C.Z., M.S.B., D.T., H.H., A.N.D., C.M., P.M., and Y.Y. C.Z. drafted the paper with subsequent comments and review by E.A.K. and A.N.D. All authors read and approved the final manuscript.

The author responsible for distribution of materials integral to the findings presented in this article in accordance with the policy described in the Instructions for Authors (https://academic.oup.com/plphys/pages/general-instructions) is: Elizabeth A. Kellogg (ekellogg@danforthcenter.org).
