# Supplementary material for: Pleiotropic and nonredundant effects of an auxin importer in Setaria and maize
Source: Plant Physiol. 2022 Mar 14;189(2):715–34. doi: 10.1093/plphys/kiac115 (PMC9157071; doi:10.1093/plphys/kiac115)
Supplement: kiac115_Supplementary_Data [file kiac115_supplementary_data.zip › Supplemental Figure legends with editors comments.pdf]

Supplemental Figure legends with editor's comments.

Figure S1. Additional phenotypes of *spp1* mutant plants. (A-G) Shoot phenotypes comparing wildtype (A10.1, white box) with *spp1* mutants (gray box). (A) Days to heading. (B) Days to anthesis. (C) Size of upper (fertile) floret (mm). (D) Percent seed germination. (E) Peduncle diameter. (F, G) Cross sections of peduncles stained with toluidine blue. (F) wildtype (A10.1); (G) *spp1*. Arrows, vascular bundles; scale bar = 200 µm. (H-N) Root phenotypes comparing wildtype (A10.1, white box) with *spp1* mutants (gray box). (H, I) Density of root hairs on the main root (H) and lateral roots (I). (J, K). Number of root hair initials on the main root (J) and lateral roots (K). (L, M) Main and lateral roots of wildtype (L) and *spp1* (M) showing differences in root hair density. Scale =2 mm. (N) Washed root systems of wildtype (left) and *spp1* (right) showing similar sizes. Scale = 1 cm. Boxes extend from lower quartile boundary to upper quartile; horizontal bar is median. Whiskers extend to the smallest and largest values within 1.5 times the interquartile range. Dots indicate individual data points. Significance values determined by Welch's t-test. = square, 0.01-0.05, *, <0.01, **, <0.001, ***, <0.0001. Mean, standard deviation (s.d.), sample sizes, and p values in Table S1.

Commented [PE1]: Please move titles (only) to the Supplemental Data section above.

Please delete the full supplemental legends over here once you have incorporated any edits in the supplemental files. Only the titles should be listed at the end of the Materials and Methods section.

In your supplemental files, please change the figure/table/dataset/movie labels to "Supplemental" with an "S" before the number (e.g., Supplemental Figure S1). The copyeditors will update the call-outs in your main manuscript file during production.

Commented [MOU2R1]: Done.

Commented [PE3]: Panels A and B show a black square in the upper right corner. Should these be asterisks? If not, please define the squares in the legend.

Commented [KE4R3]: Done.

Figure S2. Auxin rescue experiments. (A-F) Root growth and gravitropism of A10.1 (A, C and E) and *spp1* (B, D and F) at mock (A and B), 0.1 µm 2,4-D (C and D) and 0.1 µm NAA (E and F) treatments. Scale bar = 3 cm. (G-L) Root hairs of A10.1 (G, I and K) and *spp1* (H, J and L) at mock (G and H), 0.1 µm 2,4-D (I and J) and 0.1 µm NAA (K and L) treatments. Scale bar = 1 mm. (M) Root hair density on the primary roots in A10.1 and *spp1* with different auxin treatments. Box plots as in Figure S1. Significance assessed by ANOVA and Tukey's HSD. Boxes with the same letter are not significantly different at p<0.05. Mean, s.d., sample sizes, and p values in Table S1.

Commented [PE5]: I do not see the scale bar in any of the images. Please ensure the bar is clearly visible.

Commented [MOU6R5]: Corrected.

Commented [PE7]: Again, please ensure the scale bar is clearly visible.

Commented [MOU8R7]: Corrected.

Commented [PE9]: Please state the p-value used to determine significance.

Figure S3. Phenotype of *zmaux1vt2* double mutants. (A) Representative whole plant pictures. (B) Ear row number. (C) Spikelets per row. (D) Total number of kernels. (E) Number of tassel branches. (F) Number of spikelets on the main spike of the tassel. (G) Number of spikelets per cm (spikelet density). (H) Tassel length (cm). (I) Flag leaf height (cm) from ground. (J) Total number of leaves. Branch number, tassel spikelet number per cm and kernel number measured

at 56 DAS. Left to right, WT (white box), *zmaux1* (light gray box), *vt2* (dark gray box), *zmaux1vt2* (black box). Box plots as in Figure S1. Significance assessed by ANOVA and Tukey's HSD. Boxes with the same letter are not significantly different at $p<0.05$. Mean, s.d., sample sizes, and p values in Table S3.

Commented [PE10]: Please state the p-value used to determine significance.

Commented [KE11R10]: Done.

Figure S4. Phenotype of *zmaux1bif2* double mutants. (A) Representative whole plant pictures. (B) Ear row number. (C) Spikelets per row. (D) Total number of kernels. (E) Number of tassel branches. (F) Number of spikelets on the main spike of the tassel. (G) Number of spikelets per cm (spikelet density). (H) Tassel length (cm). (I) Flag leaf height (cm) from ground. (J) Total number of leaves. Branch number, tassel spikelet number per cm and kernel number measured at 56 DAS. Left to right, WT (white box), *zmaux1* (light gray box), *bif2* (dark gray box), *zmaux1bif2* (black box). Box plots as in Figure S1. Significance assessed by ANOVA and Tukey's HSD. Boxes with the same letter are not significantly different at $p<0.05$. Mean, s.d., sample sizes, and p values in Table S3.

Commented [PE12]: Please state the p-value used to determine significance.

Figure S5. Phenotype of *zmaux1Bif4* double mutants. (A) Representative whole plant pictures. (B) Ear row number. (C) Spikelets per row. (D) Total number of kernels. (E) Number of tassel branches. (F) Number of spikelets on the main spike of the tassel. (G) Number of spikelets per cm (spikelet density). (H) Tassel length (cm). (I) Flag leaf height (cm) from ground. (J) Total number of leaves. Branch number, tassel spikelet number per cm and kernel number measured at 56 DAS. Left to right, WT (white box), *zmaux1* (light gray box), *Bif4* (dark gray box), *zmaux1Bif4* (black box). Box plots as in Figure S1. Significance assessed by ANOVA and Tukey's HSD. Boxes with the same letter are not significantly different at $p<0.05$. Mean, s.d., sample sizes, and p values in Table S3.

Commented [PE13]: Please state the p-value used to determine significance.

Figure S6. Cellular localization of SPP1~iGFP. (A) Schematic diagram of SPP1 protein topology showing hydrophilic regions predicted to be in extra- and intracellular spaces. Green arrow indicates the position of GFP inserted in the N-terminal cytoplasmic loop (internal) to test SPP1~iGFP localization. Closed blue circle indicates the position of Phe$_{377}$ to Leu$_{377}$ substitution in the *spp1-C* gene. (B-D) Confocal images of *N. benthamiana* leaf cells transiently expressing SPP1~iGFP, showing localization to a thin line around the cell. Panels from left to right:

Commented [PE14]: Please consider using a color combination other than red/green, as an aid to colorblind readers.

Commented [KE15R14]: Unfortunately this is a standard fluorophore (green fluorescent protein) and a standard wavelength for showing chlorophyll autofluorescence (red). We think that replacing these standard and widely understood colors would be misleading for the majority of readers, although you are right that they are difficult for colorblind readers.

SPP1~iGFP (B), chlorophyll autofluorescence (C), and overlay (D). Scale = 20 µm. (E-J) Stable expression of SPP1~iGFP in roots of *S. viridis* at 9 DAS. Imaging of root tissues focused on either inner (E, F) or outer tissues (G-J) showing fluorescent signals on the plasma membrane (PM), predominantly in the epidermis. (I) Enlarged image of the boxed region of (H), confirming GFP signals around the nuclear membrane (yellow arrowheads). (J) Non-transgenic control.

Figure S7. Validation of SPP1~iGFP in transgenic *S. viridis*. (A) Gel image of PCR results confirming the presence of GFP band (~188bp, bottom bands) in transgenic *S. viridis* plants. PCR bands at ~540bp correspond to the *S. viridis* gene (*Sevir.2G209800*) serving as a positive control. (B) RT-qPCR assay determining the expression of SPP1~iGFP in transgenics. (C-K) Expression of SPP1~iGFP partially rescued the *spp1* defects in inflorescence and roots. Box plots, left to right A10.1 (white box), *spp1*_NT (light gray box), *spp1*_T (dark gray box). (C) Representative plants from A10.1, non-transgenic (*spp1_NT*) and transgenic (*spp1_T)* lines at 26 DAS. (D) Plant height at 23, 34, and 40 DAS for the three genotypes. (E) Representative panicles from A10.1, non-*spp1_NT* and *spp1_T* plants at 30 DAS. (F-I) Inflorescence traits for all three genotypes at 35 DAS. (F) Panicle length. (G) Primary branch number. (H) Spikelet number per branch. (I) Bristle number per branch. (J) Root growth assay showing agravitropic response of *spp1_T* seedlings at 5 DAS. (K) Percentage of agravitropic seedlings in wt and transgenics. Box plots as in Figure S1. Significance assessed by ANOVA and Tukey's HSD. Boxes with the same letter are not significantly different at $p<0.05$. Mean, s.d., sample sizes, and p values in Table S4. Scale bars = 2 cm (C), 1 cm (E, J).

Figure S8. Gene co-expression modules. Weighted gene correlation network analysis (WGCNA) detected seven co-expression modules in wild *S. viridis* A10.1. (A) Cluster dendrogram shows co-expression module assignment. (B) Expression patterns of module genes and module eigengene are shown by heatmap (top) and bar graph (bottom), respectively.

Figure S9. Comparisons between wild *S. viridis* A10.1 and *spp1* mutant networks. (A) Preservation analysis of WGCNA modules in the reference genotype (wildtype *S. viridis* A10.1) versus the test genotype (*spp1* mutant) and conversely (B). Zsummary>10, high preservation, 2< Zsummary<10, weak to moderate preservation, Zsummary<2, no preservation. (C) Similarity

Commented [PE16]: Please ensure the scale bar is correct. It seems to be the same as in H although showing an enlarged image.

Commented [KE17R16]: Thanks for catching that. The bar in I was larger than the one in H but not by enough. It has been replaced.

Commented [PE18]: Please provide the scale bar length.

Commented [KE19R18]: Done.

Commented [PE20]: Please provide the scale bar length.

Commented [KE21R20]: Done.

Commented [PE22]: Please provide the scale bar length.

Commented [KE23R22]: Done.

Commented [PE24]: Please state the p-value used to determine significance.

Commented [MOU25R24]: Added.

Commented [PE26]: Please check that all text in this figure is legible at the printed size.
Text size increased.

Commented [PE27]: Please also provide details for the mutant portion of the figure.
Added.

analysis using numbers of overlapping genes in WGCNA modules between genotypes, showing the number of overlapping genes and p-values from Fisher's exact test (in parentheses). White to red color gradient indicates –log10(p-value).

Figure S10. GO enrichment. GO enrichment analysis of major WGCNA modules in *S. viridis* A10.1, and *spp1* mutant. Dot color represents statistical significance of the enrichment (adjusted p-value, a color gradient from blue (<0.01) to red (<0.05)). The sizes of the dots represent gene ratio (number of significant genes/number of annotated genes in each GO term). GO terms were not significantly enriched in red and grey modules in A10.1, and grey module in *spp1* (not displayed).

Figure S11. Chord diagram illustrating how WGCNA module membership differs between genotypes. Color keys on the left side of the diagram represent the seven modules identified by WGCNA in wild *S. viridis* A10.1, and on the right side represent the ten modules in *spp1*. Paths of reassignment of genes are illustrated as flows in the diagram. The inner color keys ring of wild *S. viridis* A10.1 (left half) represents the reassigned modules in the *spp1* mutant. The three dashed lines show changes of module membership of five auxin-related genes.

Figure S12. Mutants of auxin importer genes. (A) Target sequences for gRNA1 (cyan arrow) and gRNA2 (magenta arrow), respectively. Boldface letters represent the PAM sites. On the gene models for the five auxin importer genes in *S. viridis*, SPP1, SvAUX2-SvAUX5, cyan and magenta arrows show locations of target sites. Numbers at ends of arrows indicate number of mismatches between gRNA and target sites. (B) Table of edits at gRNA target sites one and two in each of the five auxin influx carrier genes in each line. x, no editing; +, addition; -, deletion; bp, base pair; ->, substitution. (C) Roots of ME034V, *spp1-C,aux5* and *spp1-C,aux2,5* in 7 DAS plants. (D,E) Box plots in the same plants as in (C); left to right Me034V (white box), *spp1-C,aux5* (light gray box), and *spp1-C,aux2,5* (dark gray box). (D) Lateral root number and (E) primary root length. Box plots as in Figure S1. Significance assessed by ANOVA and Tukey's HSD. Boxes with the same letter are not significantly different at p.<0.05. Mean, s.d., sample sizes, and p values in Table S9.

Commented [PE28]: Please state the p-value used to determine significance.

Video S1. Confocal 3D reconstruction of a single inflorescence meristem similar to that shown in Figure 6G for Setaria SPP1~iGFP expression domains.

Video S2. Confocal 3D reconstruction of early development of a single inflorescence corresponding to that shown in Figure 6I for Setaria SPP1~iGFP expression domains. Image shows primary and some secondary branch meristems.

Video S3. Confocal 3D reconstruction of a single vegetative shoot apical meristem showing no expression of Setaria SPP1~iGFP

Table S1. Phenotypic comparisons between A10.1 and *spp1* mutants.

Table S2. Phenotypic comparisons between A10.1 and *spp1* mutants over development.

Table S3. Phenotypic comparisons between W22 (maize wildtype), *zmaux1* and single and double mutants of selected genes in the auxin pathway.

Table S4. Phenotypic comparisons between A10.1, spp1_T and spp1_NT, testing for complementation of SPP1~GFP.

Table S5. RNA-seq library sequencing and mapping statistics.

Table S6. Expression of all *S. viridis* genes from each replicate (R1-R4) of the developmental stages (10, 12, 14 DAS) in A10.1 and *spp1*.

Table S7. Expression of differentially expressed genes between A10.1 and *spp1* at each developmental stage (10, 12, 14 DAS).

Table S8. Expression of auxin pathway related genes in A10.1 and *spp1* at each developmental stage (10, 12, 14 DAS).

Table S9. Phenotypic comparisons among AUX CRISPR mutants.

Table S10: Primers used in this study.

Commented [PE29]: Please provide the legends for the supplemental movies in a separate PDF titled "Supplemental Movie Legends".

Commented [PE30]: Please ensure all videos play correctly. This video is in a different format from the others and did not play correctly for me.

Commented [PE31]: Please combine supplemental tables 1-6 and 8-10 into a single XLSX file (with each table fully labeled in the first row and numbered in the tab at the bottom of the page). Present each table on a separate sheet.

Commented [MOU32R31]: Done.
