# Supplementary material for: Pleiotropic and nonredundant effects of an auxin importer in Setaria and maize
Source: Plant Physiol. 2022 Mar 14;189(2):715–34. doi: 10.1093/plphys/kiac115 (PMC9157071; doi:10.1093/plphys/kiac115)
Supplement: kiac115_Supplementary_Data [file kiac115_supplementary_data.zip › Supplemental Movie Legends.pdf]

Supplemental Movie Legends

Supplemental Videos S1-S3. Localization of SPP1~iGFP in stably transformed *Setaria* inflorescences at 11 days after sowing (DAS; S1, S2) and shoot apical meristem at 6 DAS (S3).

Supplemental Video S1. Apex of the inflorescence meristem (IM) and two lateral branch primordia, showing localization of SPP1~iGFP signal (green). SPP1~iGFP expression is absent the apex of the IM. Merged image of green (GFP signals) and magenta (FM4-64 signals) channels. Meristem at the same stage as that of Figure 6G.

Supplemental Video S2. Apex of the inflorescence meristem and several branch primordia, showing epidermal enrichment of SPP1~iGFP expression in meristems of elongating primary branches. A few secondary branches also express SPP1~iGFP. SPP1~iGFP expression is absent the apex of the IM and in older branch meristems. Meristem at the same stage as that of Figure 6H. IM lacks fluorescent signals. Merged image of green (GFP signals) and magenta (for FM4-64 signals) channels.

Supplemental Video S3. Apex of the vegetative shoot apical meristem and leaf primordia. No signal is visible from SPP1~iGFP in the meristem or primordia. Some very restricted signal is visible in a few vascular cells and leaf tips but this is not reproducible.
