# Supplementary material for: Pleiotropic and nonredundant effects of an auxin importer in Setaria and maize
Source: Plant Physiol. 2022 Mar 14;189(2):715–34. doi: 10.1093/plphys/kiac115 (PMC9157071; doi:10.1093/plphys/kiac115)
Supplement: kiac115_Supplementary_Data [file kiac115_supplementary_data.zip › Supplemental_Figures_S1-S12.pdf]

Supplemental Figure S1

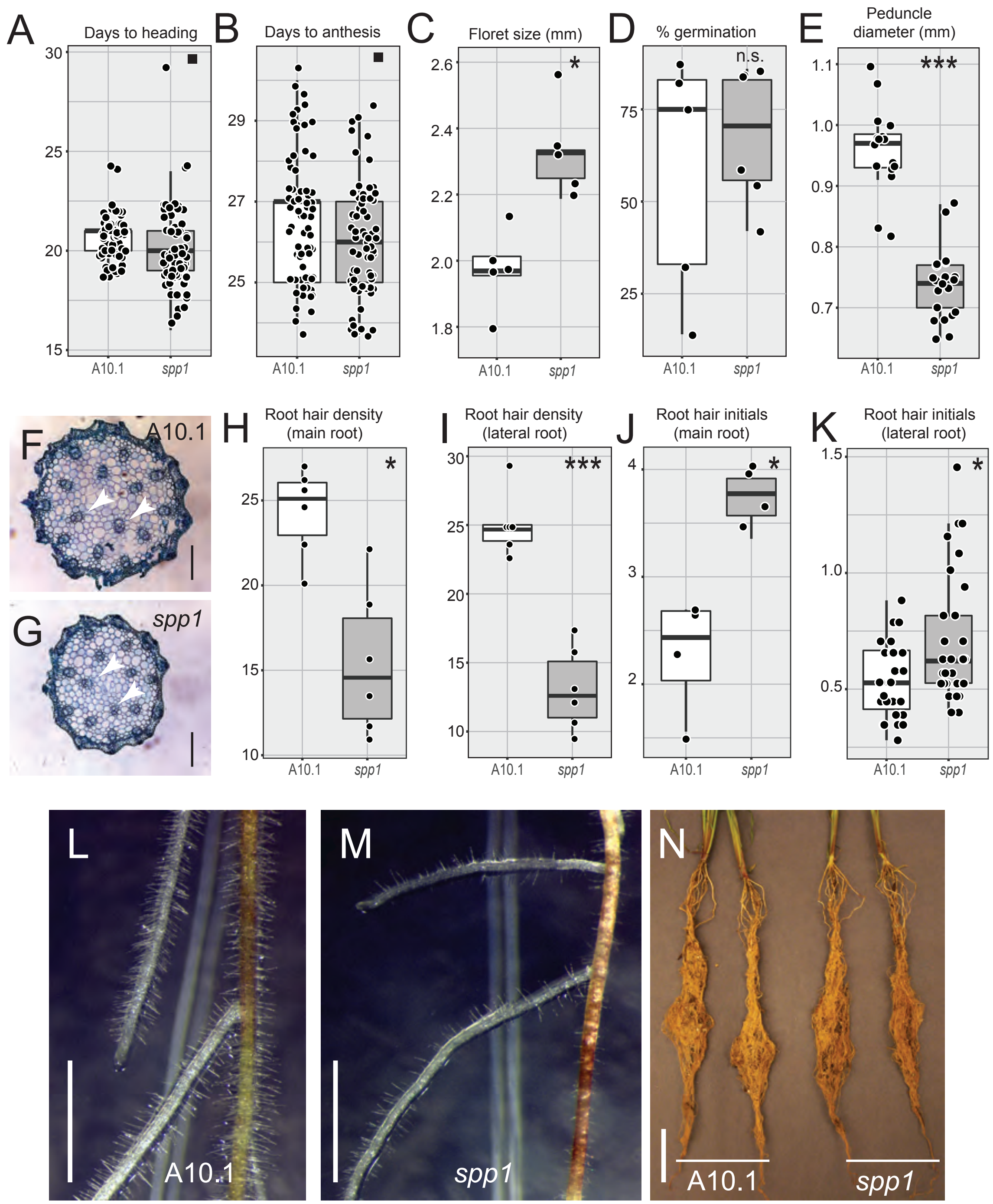

Supplemental Figure S1. Additional phenotypes of *spp1* mutant plants. (A-G) Shoot phenotypes comparing wildtype (A10.1, white box) with *spp1* mutants (gray box). (A) Days to heading. (B) Days to anthesis. (C) Size of upper (fertile) floret (mm). (D) Percent seed germination. (E) Peduncle diameter. (F, G) Cross sections of peduncles stained with toluidine blue. (F) wildtype (A10.1); (G) *spp1*. Arrows, vascular bundles; scale bar = 200 μm. (H-N) Root phenotypes comparing wildtype (A10.1, white box) with *spp1* mutants (gray box). (H, I) Density of root hairs on the main root (H) and lateral roots (I). (J, K). Number of root hair initials on the main root (J) and lateral roots (K). (L, M) Main and lateral roots of wildtype (L) and *spp1* (M) showing differences in root hair density. Scale =2 mm. (N) Washed root systems of wildtype (left) and *spp1* (right) showing similar sizes. Scale = 1 cm. Boxes extend from lower quartile boundary to upper quartile; horizontal bar is median. Whiskers extend to the smallest and largest values within 1.5 times the interquartile range. Dots indicate individual data points. Significance values determined by Welch's t-test. square, 0.01-0.05, *, <0.01, **, <0.001, ***, <0.0001. Mean, standard deviation (s.d.), sample sizes, and p values in Table S1.

Supplemental Figure S2

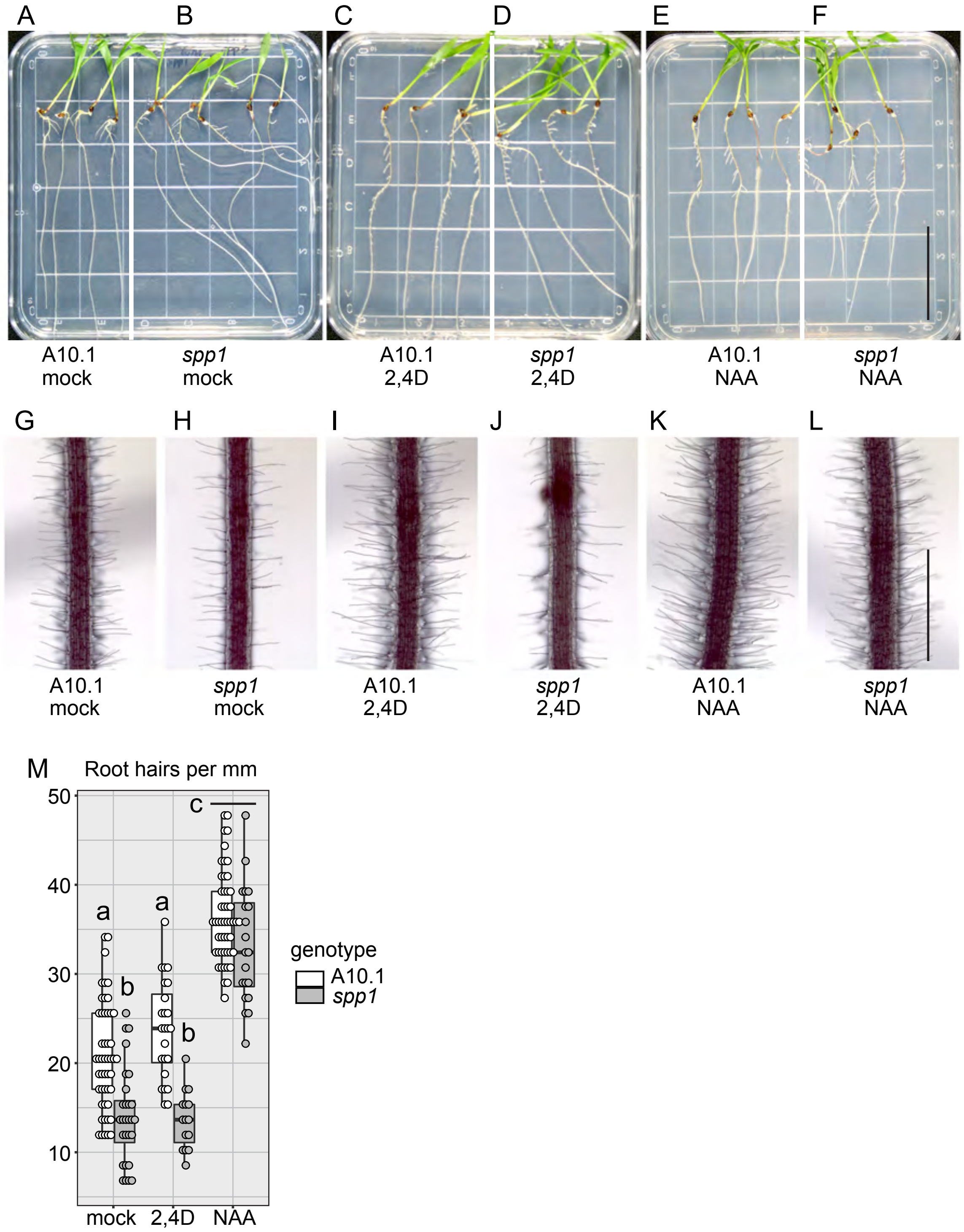

Supplemental Figure S2. Auxin rescue experiments. (A-F) Root growth and gravitropism of A10.1 (A, C and E) and *spp1* (B, D and F) at mock (A and B), 0.1 µm 2,4-D (C and D) and 0.1 µm NAA (E and F) treatments. Scale bar = 3 cm. (G-L) Root hairs of A10.1 (G, I and K) and *spp1* (H, J and L) at mock (G and H), 0.1 µm 2,4-D (I and J) and 0.1 µm NAA (K and L) treatments. Scale bar = 1 mm. (M) Root hair density on the primary roots in A10.1 and *spp1* with different auxin treatments. Box plots as in Figure S1. Significance assessed by ANOVA and Tukey's HSD. Boxes with the same letter are not significantly different at p<0.05. Mean, s.d., sample sizes, and p values in Table S1.

Supplemental Figure S3

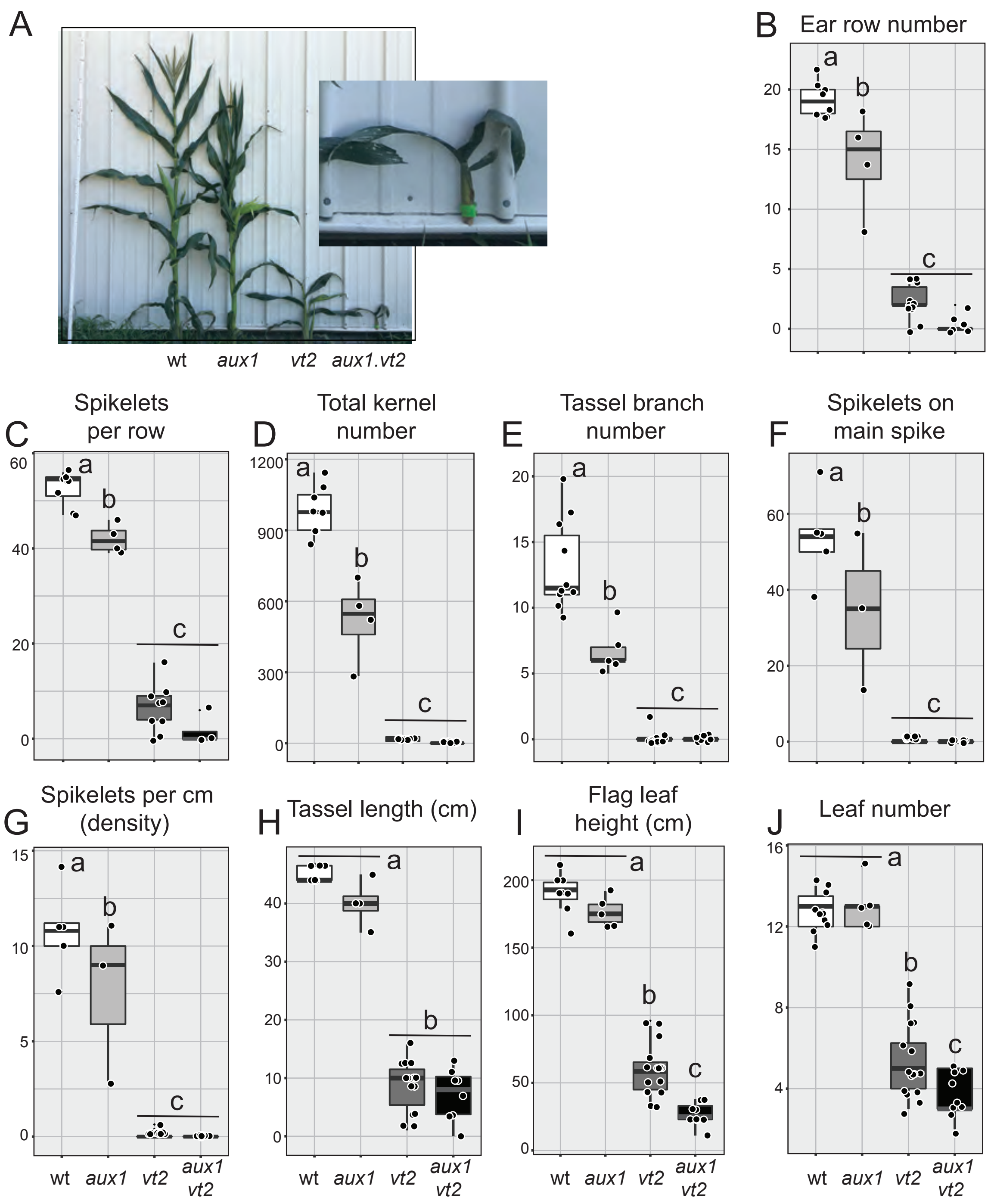

Supplemental Figure S3. Phenotype of *zmaux1vt2* double mutants. (A) Representative whole plant pictures. (B) Ear row number. (C) Spikelets per row. (D) Total number of kernels. (E) Number of tassel branches. (F) Number of spikelets on the main spike of the tassel. (G) Number of spikelets per cm (spikelet density). (H) Tassel length (cm). (I) Flag leaf height (cm) from ground. (J) Total number of leaves. Branch number, tassel spikelet number per cm and kernel number measured at 56 DAS. Left to right, WT (white box), *zmaux1* (light gray box), *vt2* (dark gray box), *zmaux1vt2* (black box). Box plots as in Figure S1. Significance assessed by ANOVA and Tukey's HSD. Boxes with the same letter are not significantly different at $p<0.05$. Mean, s.d., sample sizes, and p values in Table S3.

Supplemental Figure S4

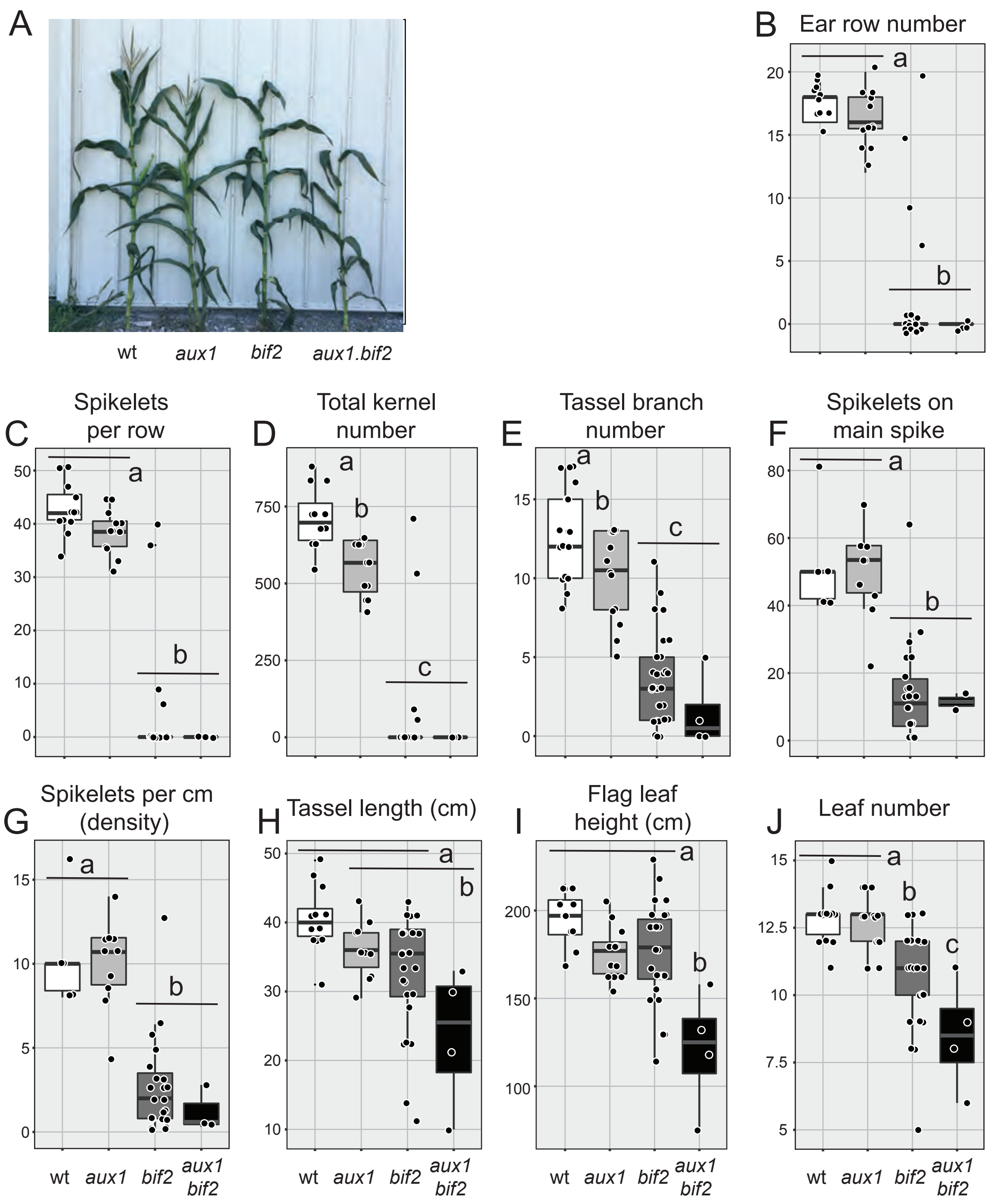

Supplemental Figure S4. Phenotype of *zmaux1bif2* double mutants. (A) Representative whole plant pictures. (B) Ear row number. (C) Spikelets per row. (D) Total number of kernels. (E) Number of tassel branches. (F) Number of spikelets on the main spike of the tassel. (G) Number of spikelets per cm (spikelet density). (H) Tassel length (cm). (I) Flag leaf height (cm) from ground. (J) Total number of leaves. Branch number, tassel spikelet number per cm and kernel number measured at 56 DAS. Left to right, WT (white box), *zmaux1* (light gray box), *bif2* (dark gray box), *zmaux1bif2* (black box). Box plots as in Figure S1. Significance assessed by ANOVA and Tukey's HSD. Boxes with the same letter are not significantly different at $p<0.05$. Mean, s.d., sample sizes, and p values in Table S3.

Supplemental Figure S5

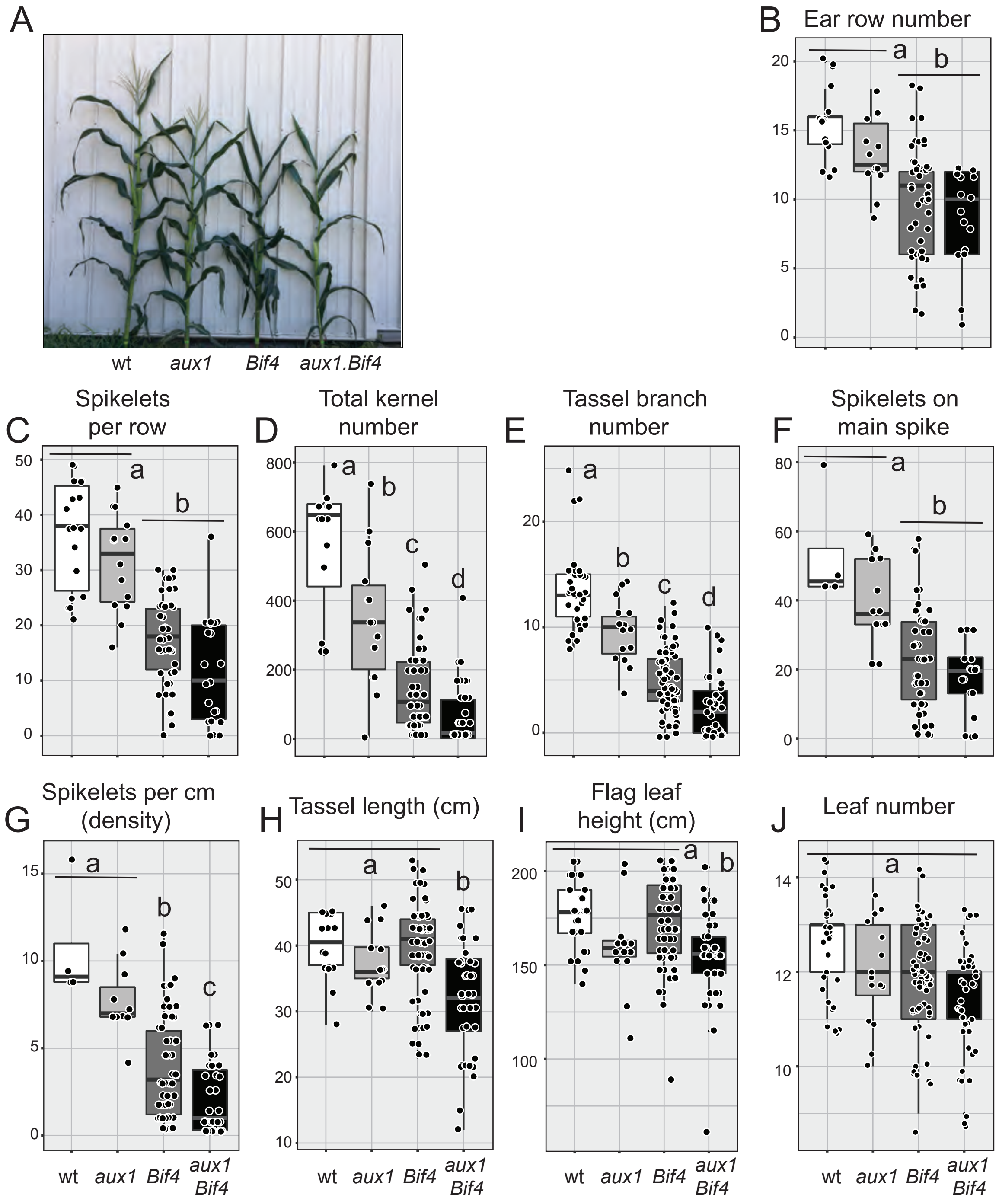

Supplemental Figure S5. Phenotype of *zmaux1Bif4* double mutants. (A) Representative whole plant pictures. (B) Ear row number. (C) Spikelets per row. (D) Total number of kernels. (E) Number of tassel branches. (F) Number of spikelets on the main spike of the tassel. (G) Number of spikelets per cm (spikelet density). (H) Tassel length (cm). (I) Flag leaf height (cm) from ground. (J) Total number of leaves. Branch number, tassel spikelet number per cm and kernel number measured at 56 DAS. Left to right, WT (white box), *zmaux1* (light gray box), *Bif4* (dark gray box), *zmaux1Bif4* (black box). Box plots as in Figure S1. Significance assessed by ANOVA and Tukey's HSD. Boxes with the same letter are not significantly differentat $p<0.05$. Mean, s.d., sample sizes, and p values in Table S3.

Supplemental Figure S6

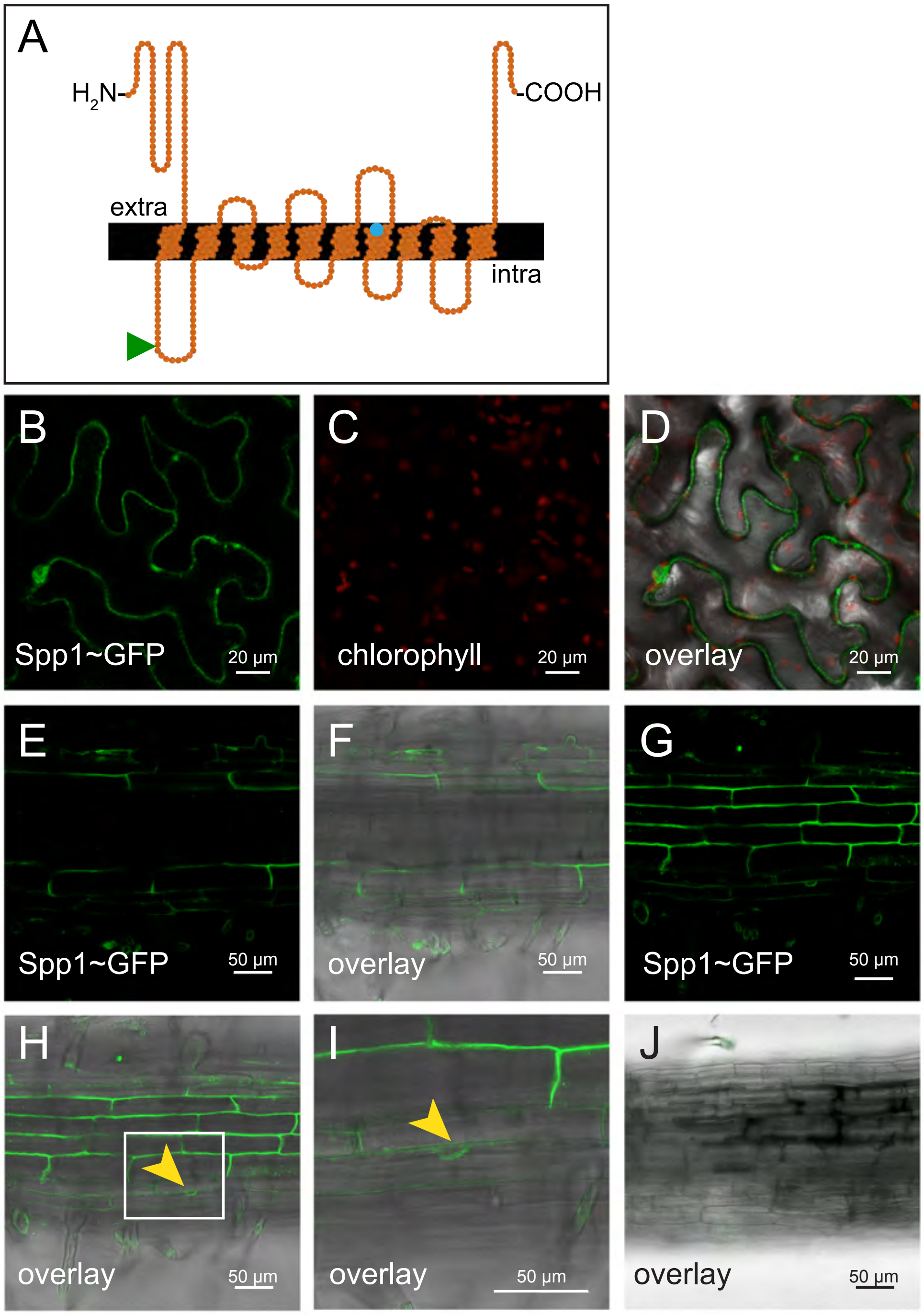

Supplemental Figure S6. Cellular localization of SPP1~iGFP. (A) Schematic diagram of SPP1 protein topology showing hydrophilic regions predicted to be in extra- and intracellular spaces. Green arrow indicates the position of GFP inserted in the N-terminal cytoplasmic loop (internal) to test SPP1~iGFP localization. Closed blue circle indicates the position of Phe377 to Leu377 substitution in the *spp1-C* gene of *spp1-C,aux2,3,4,5*. (B-D) Confocal images of cells of *Nicotiana benthamiana* leaves transiently expressing *SPP1~iGFP*, showing localization to a thin line around the cell. Panels from left to right: SPP1~iGFP (B), chlorophyll autofluorescence (C), and overlay (D). Scale = 20 µm. (E-J) Stable expression of SPP1~iGFP in roots of *S. viridis* at 9 DAS. Imaging of root tissues focused on either inner (E, F) or outer tissues (G-J) showing fluorescent signals on the plasma membrane (PM), predominantly in the epidermis. (I) Enlarged image of the boxed region of (H), confirming GFP signals around the nuclear membrane (yellow arrowheads). (J) Non-transgenic control.

Supplemental Figure S7

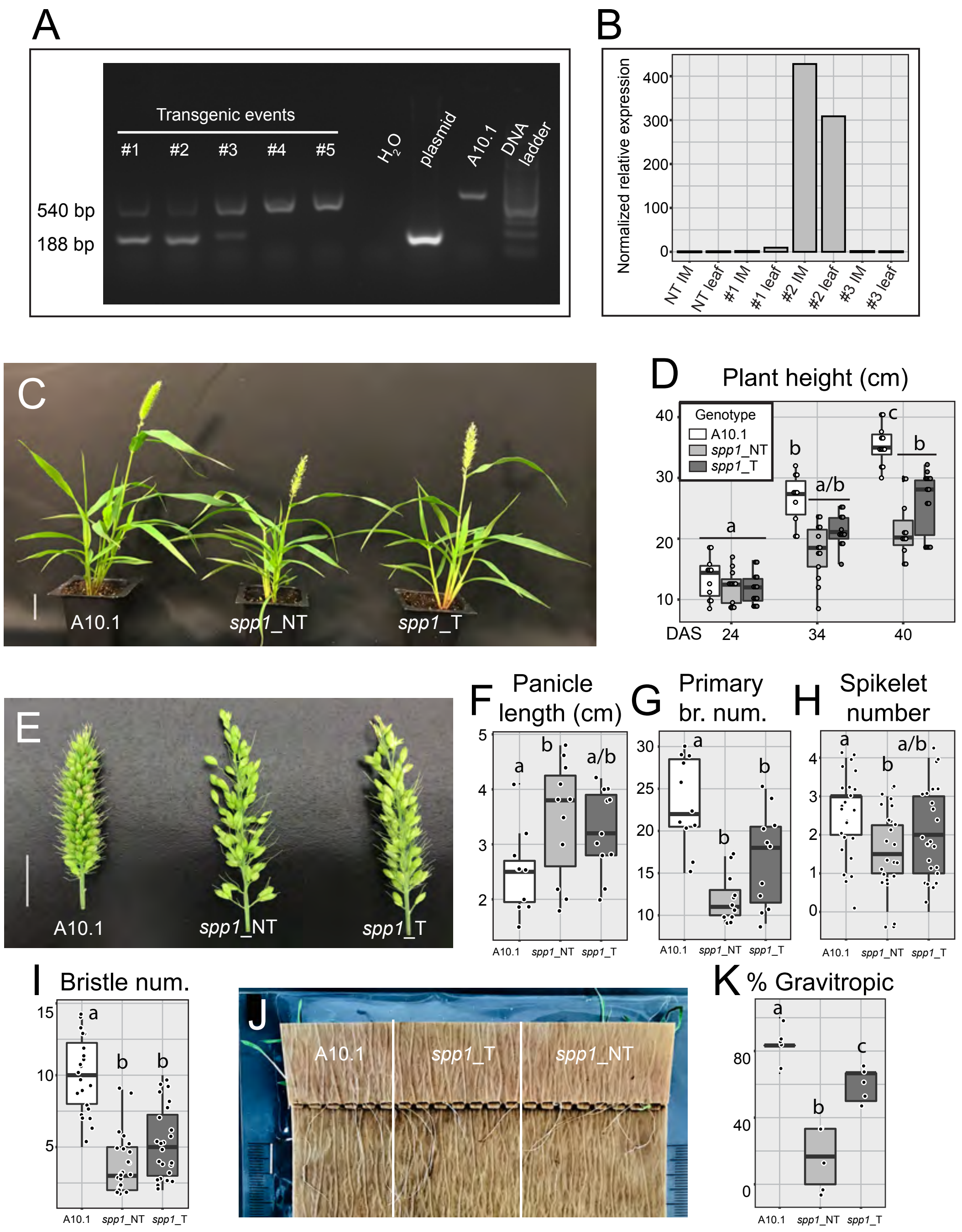

Supplemental Figure S7. Validation of SPP1~iGFP in transgenic *S. viridis*. (A) Gel image of PCR results confirming the presence of GFP band (~188bp, bottom bands) in transgenic *S. viridis* plants. PCR bands at ~540bp correspond to the *S. viridis* gene (*Sevir.2G209800*) serving as a positive control. (B) RT-qPCR assay determining the expression of SPP1~iGFP in transgenics. (C-K) Expression of *SPP1~iGFP* partially rescued the *spp1* defects in inflorescence and roots. Box plots, left to right, A10.1 (white box), *spp1_NT* (light gray box), *spp1_T* (dark gray box). (C) Representative plants from A10.1, non-transgenic (*spp1_NT*) and transgenic (*spp1_T*) lines at 26 days after sowing (DAS). (D) Plant height at 23, 34, and 40 DAS for the three genotypes. (E) Representative panicles from A10.1, *spp1_NT* and *spp1_T* plants at 30 DAS. (F-I) Inflorescence traits for all three genotypes at 35 DAS. (F) Panicle length. (G) Primary branch number. (H) Spikelet number per branch. (I) Bristle number per branch. (J) Root growth assay showing agravitropic response of spp1_T seedlings at 5 DAS. (K) Percentage of agravitropic seedlings in wt and transgenics. Box plots as in Figure S1. Significance assessed by ANOVA and Tukey's HSD. Boxes with the same letter are not significantly different at $p<0.05$. Mean, s.d., sample sizes, and p values in Table S4. Scale bars = 2 cm (C), 1 cm (E, J).

Supplemental Figure S8

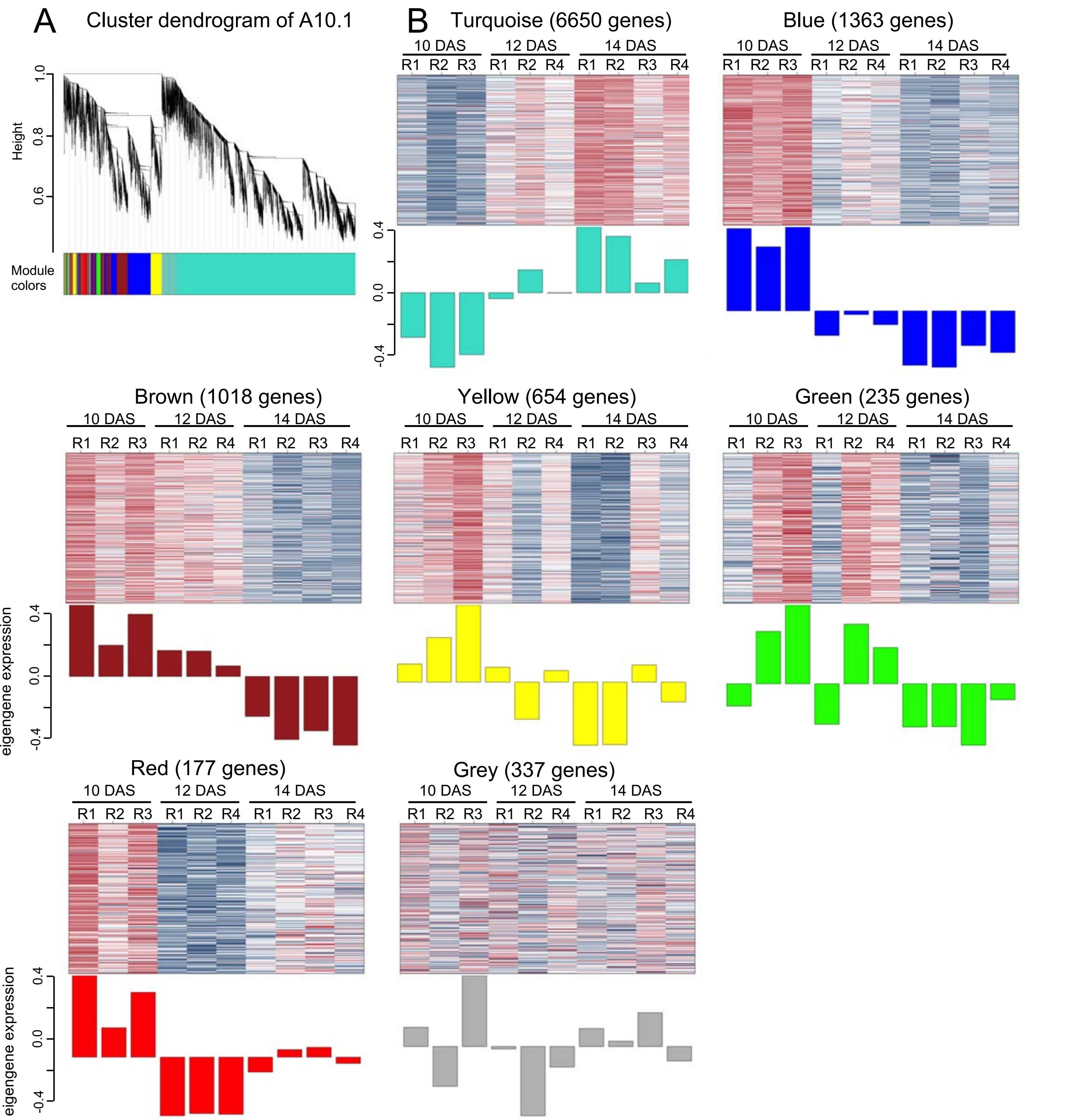

Supplemental Figure S8. Weighted gene correlation network analysis (WGCNA) for wild *S. viridis* A10.1 (A, B) and mutant *spp1* (C, D, following page). (A, C) Cluster dendrograms with co-expression module assignments, seven in A10.1 (A) and ten in *spp1* (C). (B, D) Heat map of module genes (top) and bar graphs of module eigengenes (bottom).

Supplemental Figure S8, continued

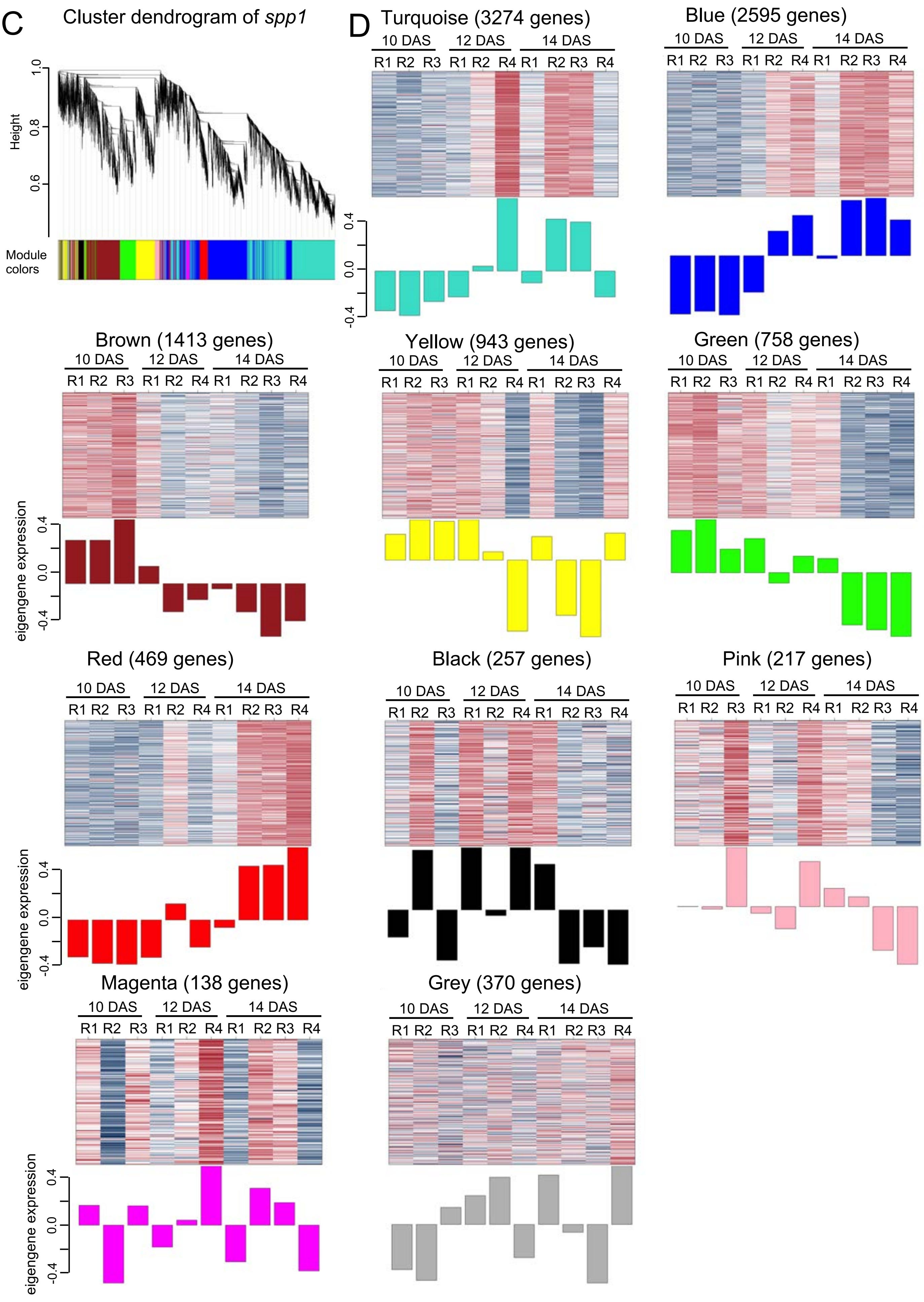

Supplemental Figure S9

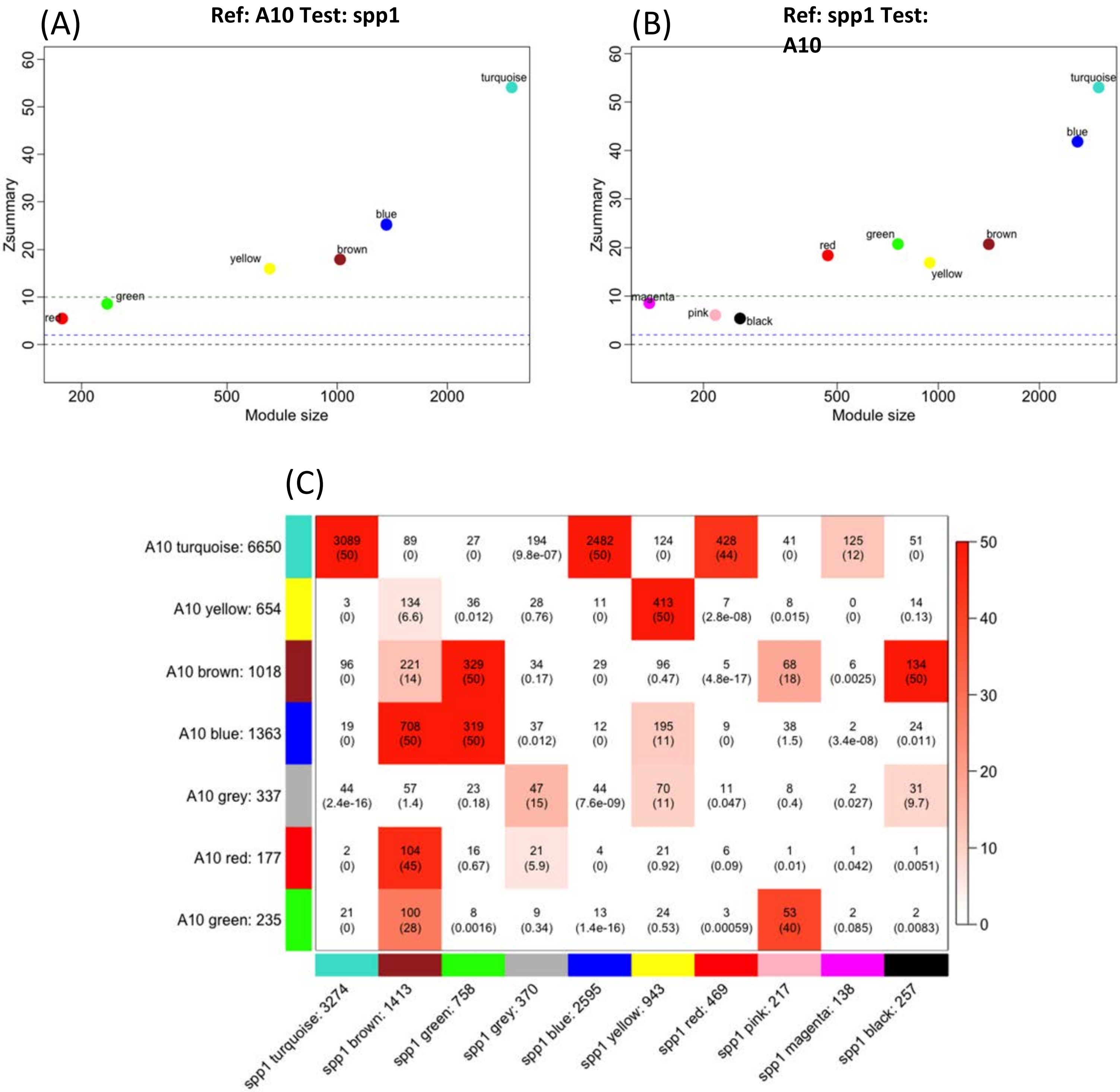

Supplement Figure S9. Comparisons between wild *S. viridis* A10.1 and *spp1* mutant networks. (A) Preservation analysis of WGCNA modules in reference genotype (wildtype *S. viridis* A10.1) versus the test genotype (*spp1* mutant) and conversely (B). Zsummary>10, high preservation, 2< Zsummary<10, weak to moderate preservation, Zsummary<2, no preservation. (C) Similarity analysis using numbers of overlapping genes in WGCNA modules between genotypes. The number of overlapping genes and p-values from Fisher's exact test (in parentheses) is shown. White to red color gradient indicates –log10(p-value).

Supplemental Figure S10

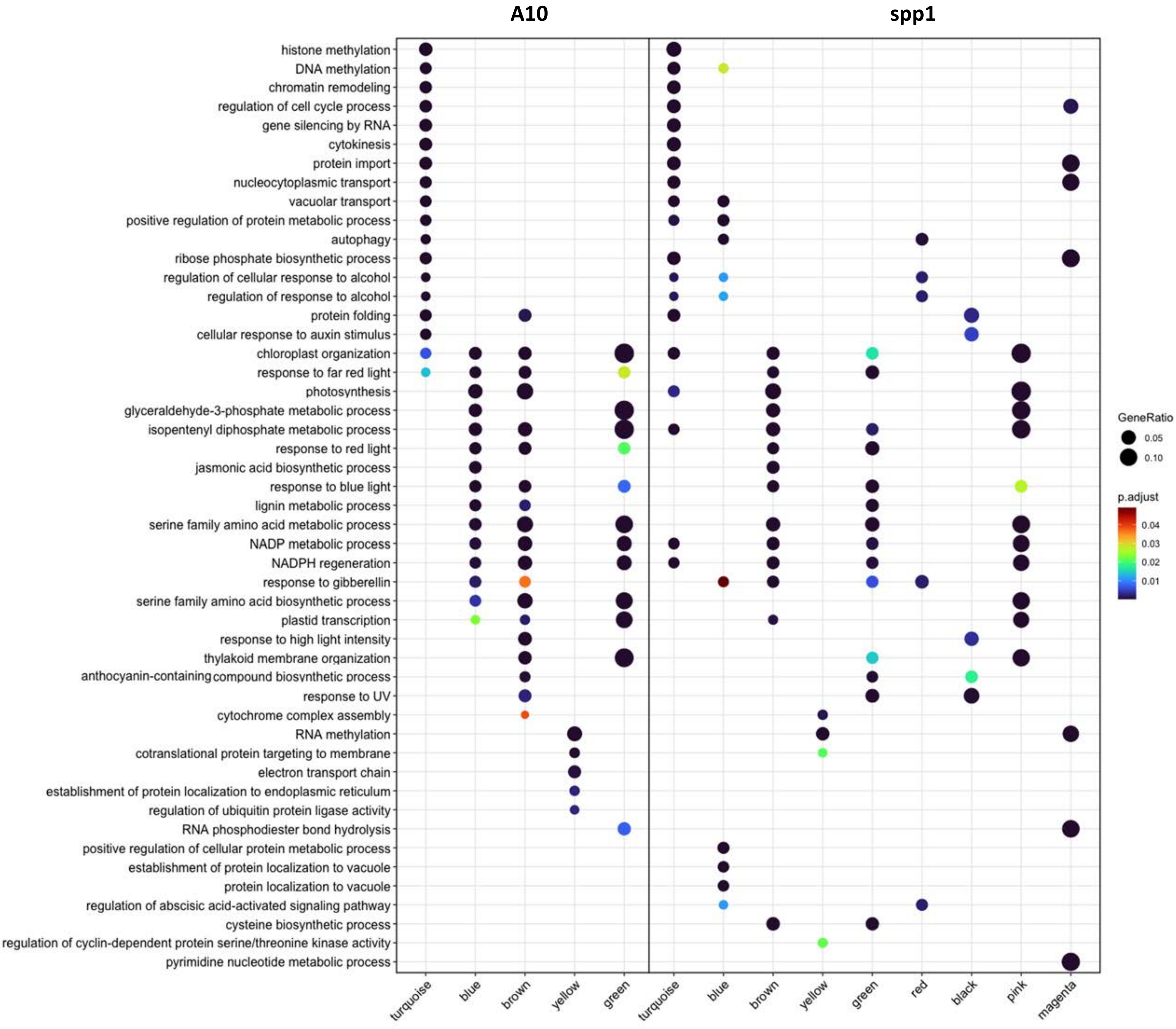

Supplemental Figure S10. GO enrichment analysis of major WGCNA modules in *S. viridis* A10.1, and *spp1* mutant. Dot color represents statistical significance of the enrichment (adjusted p-value, a color gradient from blue (<0.01) to red (<0.05)). The sizes of the dots represent gene ratio (the number of significant genes/number of annotated genes in each GO terms). GO terms were not significantly enriched in red and grey modules in A10.1, and grey module in *spp1* (not displayed).

Supplemental Figure S11

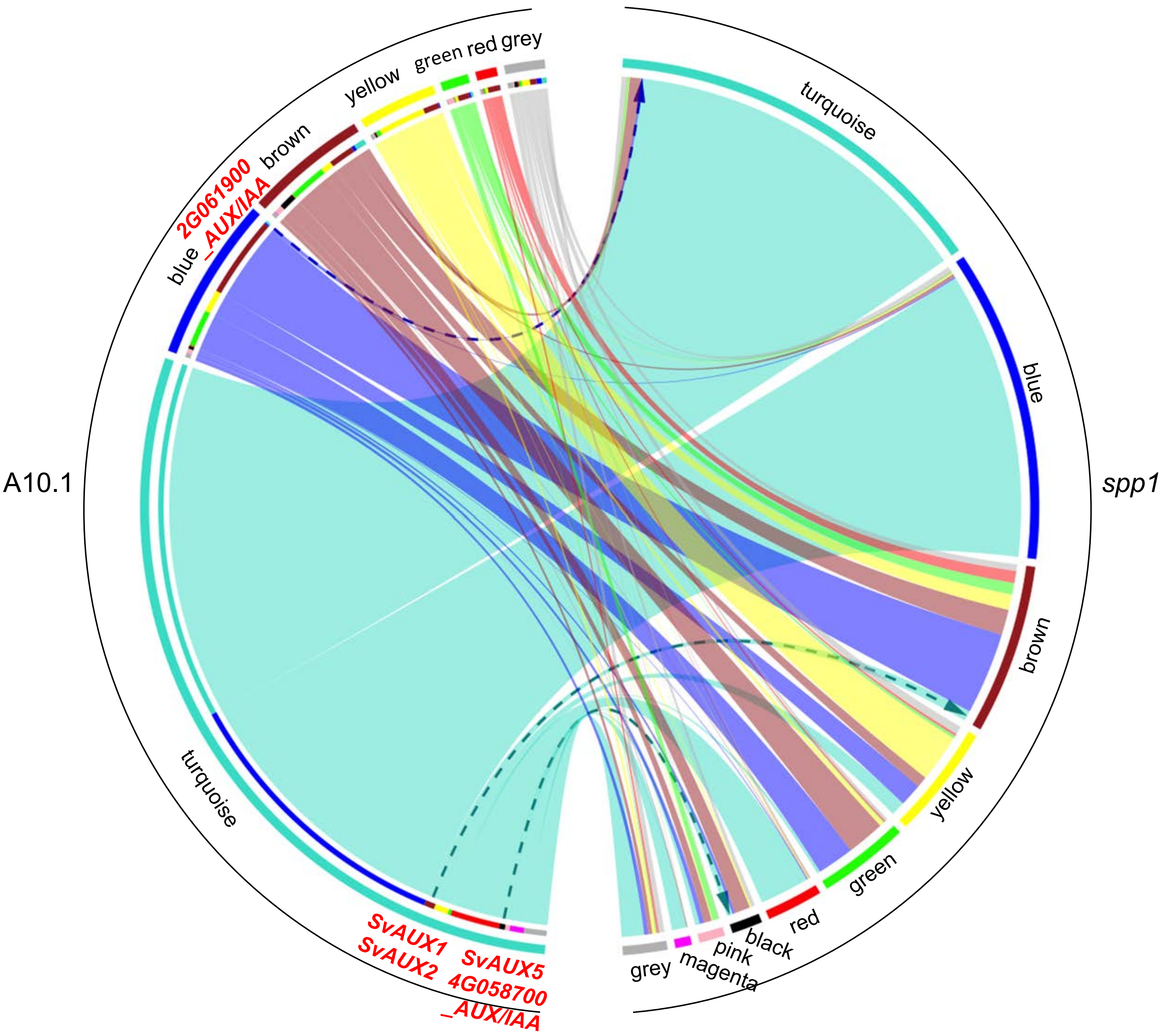

Supplemental Figure S11. Chord diagram illustrating how WGCNA module membership differs between genotypes. Color keys on the left side of the diagram represent 7 modules identified by WGCNA in wild *S. viridis* A10.1, and on the right side represent 10 modules in *spp1*. Paths of reassignment of genes are illustrated as flows in the diagram. The inner color keys ring of wild *S. viridis* A10.1 (left half) represents the reassigned modules in the *spp1* mutant. The three dashed lines show changes of module membership of five auxin-related genes.

Supplemental Figure S12

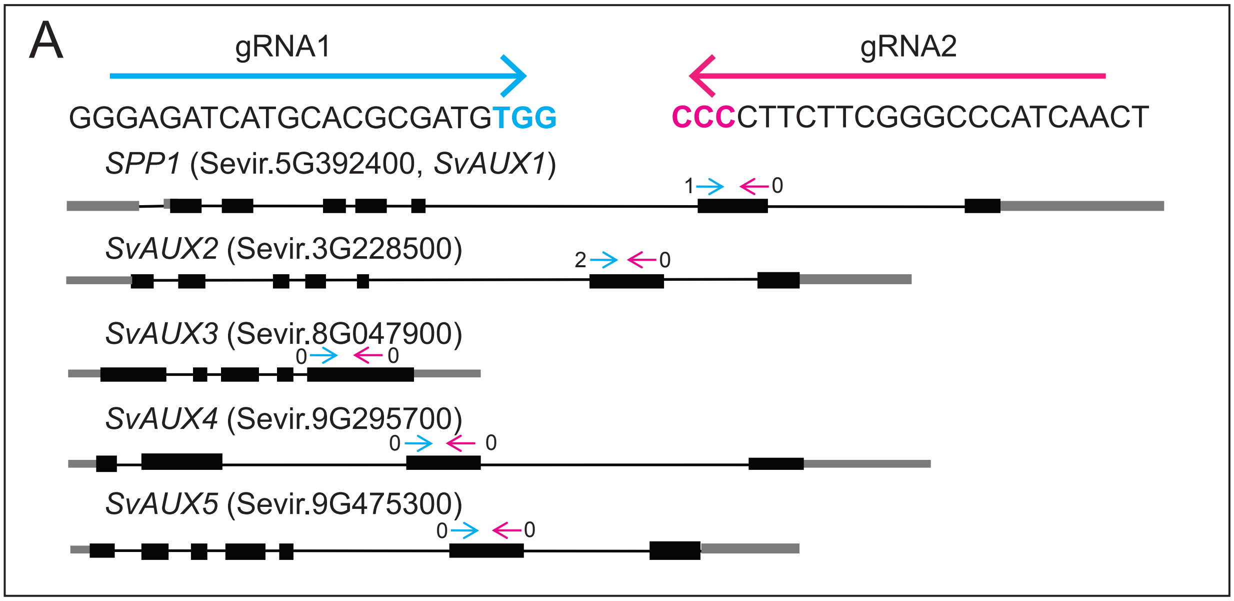

B

Line	Genotype	*SPP1*	*SvAUX2*	*SvAUX3*	*SvAUX4*	*SvAUX5*
ME034V	ME034V	(x, x)	(x, x)	(x, x)	(x, x)	(x,x)
cz66-12-21-B2-2	*spp1-C*	(x, +A)	(x, x)	(x, x)	(x, x)	(x,x)
cz66-2-N12-4-1	*spp1-C,aux5*	(x, -6bp)	(x, x)	(x, x)	(x, x)	(x, -24bp)
cz66-3-N25-4-5	*spp1-C,aux3*	(x, +A)	(x, x)	(+A, x)	(x, x)	(x,x)
cz66-4-N27-16-1	*spp1-C,aux25*	(x, -24bp)	(x, +T)	(x, x)	(x, x)	(-G, +T)
cz66-11-16-11-1-4	*aux2345*	(x, C->A)	(x, +85bp)	(-19bp, -2bp)	(x, -T)	(-G, +C)
cz66-11-17-10-2-1	*spp1-C,aux2345*	(x, -6bp)	(x,+T)	(x, +T)	(x, +T)	(x, +C)

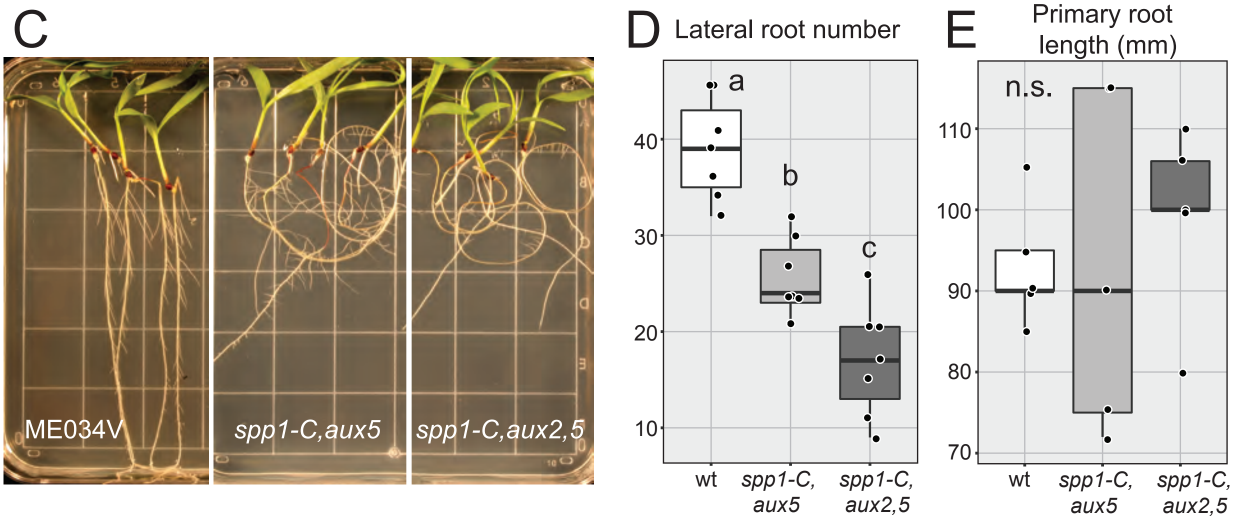

Supplemental Figure S12. Mutants of auxin importer genes. (A) Target sequences for gRNA1 (cyan arrow) and gRNA2 (magenta arrow), respectively. Boldface letters represent the PAM sites. On the gene models for the five auxin importer genes in *SPP1*, *SvAUX2-SvAUX5*, cyan and magenta arrows show locations of target sites. Numbers at ends of arrows indicate number of mismatches between gRNA and target sites. (B) Table of edits at gRNA target sites one and two in each of the five auxin influx carrier genes in each line. x, no editing; +, addition; -, deletion; bp, base pair; ->, substitution. (C) Roots of ME034V, *spp1-C,aux5* and *spp1-C,aux2,5* in 7 DAS plants. (D,E) Box plots in the same plants as in (C); left to right Me034V (white box), *spp1-C,aux5* (light gray box), and *spp1-C,aux2,5* (dark gray box). (D) Lateral root number and (E) primary root length. Box plots as in Figure S1. Significance assessed by ANOVA and Tukey's HSD. Boxes with the same letter are not significantly different at $p<0.05$. Mean, s.d., sample sizes, and p values in Table S9.
